# Morin-Loaded PLGA-Chitosan Nanoparticles Attenuate PTZ-Induced Seizure-Related Behavioral, Biochemical, and Transcriptional Changes in Male Rats

**DOI:** 10.3390/pharmaceutics18091170

**Published:** 2026-09-16

**Authors:** Ashraf Kakoo, Azad Hasan Kheder, Ali A. Mohammedsaeed, Trefa Salih Mohamad, Mohammed Awat Ali, Mohammad B. Ghayour, Arash Abdolmaleki, Dlzar B. Rahman, Shang Ziyad Abdulqadir, Taban Kamal Rasheed, Mohammed Jarjees Hashm, Shukur Wasman Smail

**Affiliations:** 1 Department of Biology, College of Science, Salahaddin University-Erbil, Erbil 44001, Iraq; ashraf.kako@su.edu.krd (A.K.); trefa.mohamad@su.edu.krd (T.S.M.); shang.abdulqadir@su.edu.krd (S.Z.A.); taban.rasheed@su.edu.krd (T.K.R.); 2 Physiotherapy Department, Erbil Health and Medical Technical College, Erbil Polytechnic University, Erbil 44001, Iraq; azad1.hasan1978@gmail.com; 3 Department of Medical Laboratory Science, College of Health Sciences, Lebanese French University, Erbil 44001, Iraq; ali.abdulhamid@lfu.edu.krd; 4 Faculty of General Medicine, Koya University, Koya 46017, Iraq; mohammedawat23@gmail.com; 5 Department of Biophysics, Faculty of Advanced Technologies, University of Mohaghegh Ardabili, Namin 56391-44991, Iran; mohammad.ghayour21@gmail.com (M.B.G.); abdolmalekiarash1364@gmail.com (A.A.); 6 Department of Genetics, Bio Diagnostic Center (BDC), Erbil 44001, Iraq; dlzarr.rahman@gmail.com (D.B.R.); mohammadjerjes8888@gmail.com (M.J.H.); 7 Department of Medical Science, Respiratory Medicine, and Allergology, Uppsala University and University Hospital, Uppsala 75185, Sweden

**Keywords:** morin, PLGA, chitosan, nanoparticles, anticonvulsant, anxiety, neuroinflammation, Nrf2, GFAP, Iba1

## Abstract

**Aims:** Morin is a flavonoid with potential neuroprotective and anti-inflammatory properties. This study evaluated the anticonvulsant and anxiolytic effects of morin-loaded PLGA-chitosan nanoparticles (Morin-PLGA-CS NPs) in male Wistar rats. **Methods:** Morin-PLGA-CS NPs were synthesized using a modified single emulsion-solvent evaporation method followed by CS coating. NPs were characterized by dynamic light scattering (DLS), scanning electron microscopy (SEM), and in vitro drug release analysis. Adult male Wistar rats received intraperitoneal injections of free morin (25 mg/kg), Morin-PLGA-CS NPs, diazepam (1 mg/kg), blank NPs, or vehicle. Behavioral assessments included the open-field test (OFT), elevated-plus maze (EPM), novel object recognition (NOR) test, and pentobarbital-induced sleep test. Anticonvulsant activity was evaluated using PTZ-induced seizure latency. Cytokine concentrations in cortical and hippocampal tissue lysates were quantified by ELISA at 12 h post-PTZ. Hippocampal relative mRNA expression of Nrf2, HO-1, GFAP, and Iba1 was quantified by quantitative real-time PCR (qRT-PCR). **Results:** Morin-PLGA-CS NPs demonstrated a hydrodynamic diameter of 221.6 nm, a zeta potential of +23.3 mV, and an encapsulation efficiency of 81%. FTIR spectroscopy showed spectral changes compatible with morin incorporation and possible hydrogen-bonding interactions. The NPs exhibited approximately 81.4% morin release over 72 h in vitro. Compared to free morin, Morin-PLGA-CS NPs increased center-zone exploration in the OFT and open-arm behavior in the EPM, but also reduced total distance traveled in the OFT, indicating that motor suppression or sedation may have contributed to the behavioral profile (*p* < 0.001). It also improved the discrimination index (DI) in the NOR test and elevated sleep duration in the pentobarbital test (*p* < 0.001). The NPs also prolonged seizure latency (137.2 s vs. 114.5 s for free morin; *p* < 0.001) and markedly reduced IL-1β, IL-6, and TNF-α levels in both the cortex and hippocampus. At the molecular level, Morin-PLGA-CS NPs were associated with significantly increased hippocampal Nrf2 and HO-1 mRNA transcript levels and decreased GFAP and Iba1 transcript levels relative to free morin and the PTZ-challenged vehicle control group. **Conclusions:** Morin-PLGA-CS NPs produced greater behavioral, anticonvulsant, inflammatory, and redox effects than free morin in male rats. Molecular data revealed changes in hippocampal mRNA expression, including increased Nrf2 and HO-1 transcripts and decreased GFAP and Iba1 transcripts. However, these transcript-level findings are preliminary and require protein-level validation.

## 1. Introduction

Epilepsy is a prevalent chronic neurological disorder affecting approximately 45.9 million people worldwide, with a prevalence of 621.5 per 100,000 population [1,2]. It is characterized by recurrent, unprovoked seizures due to abnormal synchronous neuronal activity, which imposes a significant burden on patients, healthcare systems, and society [3,4]. Despite the availability of various anti-seizure medications (ASMs), approximately one-third of people with epilepsy continue to have seizures after appropriate treatment trials [5,6]. Even among those who achieve seizure control, many face dose-limiting adverse effects, including cognitive impairment, sedation, mood disturbances, dizziness, and teratogenicity, which adversely impact quality of life and therapeutic adherence [7]. These ongoing therapeutic gaps highlight the urgent need for novel antiseizure agents with improved efficacy, safety profiles, and innovative delivery strategies to enhance drug bioavailability in the central nervous system (CNS) [8].

Natural products have long been rich sources of pharmacologically active compounds for treating neurological disorders [9,10]. Among these, flavonoids have gained attention for their wide range of biological activities and generally favorable safety profiles [11]. Morin (3,5,7,2′,4′-pentahydroxyflavone) is a flavonoid found abundantly in plants of the Moraceae family as well as in various fruits and medicinal herbs [12,13]. Preclinical studies show that morin exhibits pharmacological effects, including antioxidant, anti-inflammatory, neuroprotective, anticancer, and antidiabetic activities [14,15]. In epilepsy, evidence suggests morin has anticonvulsant effects through various mechanisms, such as enhancing Gamma-aminobutyric acid (GABA)ergic signaling, reducing glutamatergic excitotoxicity, and inhibiting neuroinflammatory pathways involving nuclear factor kappa B (NF-κB) [16,17]. Moreover, morin has been reported to prevent granule cell dispersion and neurotoxicity by suppressing mechanistic target of rapamycin complex 1 (mTORC1) signaling in a kainic acid-induced seizure model [18,19]. However, the clinical use of morin, like many flavonoids, is limited by poor physical and chemical properties, including low water solubility, extensive first-pass metabolism, rapid clearance, and ineffective crossing of the blood–brain barrier (BBB) [20,21].

In recent decades, nanomedicine has emerged as an approach to overcome pharmacokinetic barriers limiting the clinical use of many promising drugs [22,23]. Polymeric nanoparticles may improve drug solubility, protect cargo from degradation, and, depending on their composition and surface properties, may influence transport across biological barriers [24]. Among polymeric nanocarriers, poly (lactic-co-glycolic acid) (PLGA) and chitosan (CS) have established biocompatibility profiles and have been used in several biomedical and pharmaceutical applications. However, their regulatory status depends on the specific formulation, indication, and route of administration [25,26,27]. PLGA is a synthetic copolymer with adjustable degradation rates. It has been used in several approved drug-delivery products, whereas the safety and regulatory status of a specific PLGA–CS formulation remains formulation- and route-dependent [28]. CS, a natural polysaccharide made from chitin, aids drug passage through cells by temporarily opening tight junctions and enhances cellular uptake through interactions with negatively charged cell membranes [29,30]. Combining morin with PLGA-CS NPs is expected to enhance morin stability and improve release. The mucoadhesive and cationic properties of CS may improve mucosal transport and cellular uptake, potentially increasing CNS delivery [25].

Beyond behavioral and biochemical endpoints, the molecular mechanisms underlying seizure-induced hippocampal injury involve complex transcriptional responses. Following seizures, the brain mounts an antioxidant defense partly mediated by nuclear factor erythroid 2-related factor 2 (Nrf2), a master transcription factor that drives the expression of cytoprotective genes, including heme oxygenase-1 (HO-1) and superoxide dismutase (SOD) [31,32]. Nrf2 is predominantly activated in hippocampal neurons after acute pentylenetetrazol (PTZ)-induced seizures, and its downstream antioxidant targets peak within hours of the ictus [32,33]. Concurrently, seizures can induce astrocytic and microglial responses, reflected by increased expression of glial fibrillary acidic protein (GFAP) and ionized calcium-binding adapter molecule 1 (Iba1), respectively [34,35]. Previous in vitro and experimental studies suggest that morin may attenuate microglia-associated inflammatory responses and modulate Nrf2/HO-1-related signaling under inflammatory conditions [36]. However, whether nanoencapsulation further amplifies these transcriptional effects in a PTZ-induced seizure model has not been evaluated. In the present study, we extended the analysis to examine whether the observed behavioral and biochemical effects were accompanied by changes in selected hippocampal Nrf2, HO-1, GFAP, and Iba1 transcripts. To our knowledge, no previous study has evaluated whether encapsulating morin in PLGA-CS NPs enhances its anticonvulsant, anxiolytic, and sedative properties in a PTZ-induced seizure model. This study was designed to address that gap by evaluating the anticonvulsant, sedative, and anxiolytic activity of Morin-PLGA-CS NPs in adult male Wistar rats.

## 2. Materials and Methods

### 2.1. Chemicals

All chemicals and reagents were of analytical or higher grade. Morin hydrate (≥95% purity) was purchased from Merck KGaA, Darmstadt, Germany (M4008). PTZ (P6500), pentobarbital (P3761), polyvinyl alcohol (341584), PLGA (719927), and CS (448869) were purchased from Sigma-Aldrich, St. Louis, MO, USA. Diazepam was obtained from Tolid Daru Pharmaceutical Co. (Tehran, Iran) in injectable form. PTZ and pentobarbital were dissolved in physiological saline (0.9%).

### 2.2. Preparation of Morin-Loaded PLGA–CS NPs

Morin-PLGA-CS NPs were synthesized using a modified single emulsion-solvent evaporation technique with electrostatic surface coating by CS. Briefly, 50 mg of PLGA (50:50 lactide: glycolide ratio, inherent viscosity 0.4–0.6 dL/g) was dissolved in 2 mL of dichloromethane (DCM) at room temperature. Then, 5 mg of morin hydrate dissolved in 500 µL of absolute ethanol was added dropwise under continuous stirring (400 rpm, 5 min) to form a homogeneous organic phase. This organic phase was added dropwise to 10 mL of a pre-cooled (4 °C) aqueous solution of 1% (*w*/*v*) polyvinyl alcohol (PVA, MW 30,000–70,000 Da, 87–90% hydrolyzed, pH 7.4) and emulsified with a probe sonicator (UP200H, Hielscher Ultrasonics GmbH, Teltow, Germany) at 70% amplitude for 2 min in pulse mode (5 s on/3 s off) to generate an oil-in-water nanoemulsion. The nanoemulsion was stirred at 600 rpm for 4 h at room temperature under a nitrogen stream to evaporate DCM. Next, 5 mg of CS (50–190 kDa, deacetylation degree ≥ 75%) dissolved in 5 mL of 0.1 M acetic acid (pH 4.0) was added dropwise to the PLGA NP suspension (pH 5.5) under moderate stirring (500 rpm, 2 h) at a CS: PLGA weight ratio of 1:10 for surface coating. The NPs were collected by ultracentrifugation at 20,000× *g* for 30 min at 4 °C, washed three times with deionized water (pH 5.5) to remove unencapsulated morin and excess polymers, re-dispersed in 2 mL of deionized water with 5% (*w*/*v*) sucrose as a cryoprotectant, and the dispersion was frozen at −80 °C for 24 h and subsequently lyophilized under vacuum [37].

### 2.3. The Encapsulation Efficiency and Drug Loading

The encapsulation efficiency (EE%) and drug loading (DL%) of Morin-PLGA-CS NPs were determined using an indirect method. Briefly, the NP suspension was centrifuged at 15,000× *g* for 30 min at 4 °C using an ultrafiltration centrifugal filter device (Amicon Ultra, Merck Millipore, Burlington, MA, USA, MWCO 10 kDa) to separate unencapsulated morin. The concentration of unencapsulated morin in the filtrate was quantified by UV-Vis spectrophotometry at 365 nm against a standard calibration curve. All measurements were performed in triplicate. EE% and DL% were calculated using the following equations: EE% = (W_total drug_ − W_free drug_)/W_total drug_ and DL% = (W_total drug_ − W_free drug_)/W_NPs_, respectively.

### 2.4. In Vitro Drug Release Study

The in vitro release profile of morin from PLGA-CS NPs was assessed using the dialysis bag method. A number of lyophilized nanoparticles corresponding to 2 mg of loaded morin was dispersed in 2 mL of phosphate-buffered saline (PBS, pH 7.4) and placed in a pre-treated dialysis membrane (Molecular Weight Cut-Off: 12–14 kDa). The bag was immersed in 50 mL of PBS (pH 7.4) containing Tween 80 (0.5%) to maintain sink conditions and incubated at 37 ± 0.5 °C with gentle agitation at 100 rpm. At specified intervals (0.5, 1, 2, 4, 8, 12, 24, 48, and 72 h), 2 mL aliquots of the medium were taken and replaced with fresh PBS. Morin concentration was quantified by UV-Vis spectrophotometry at 365 nm. Cumulative release was corrected for the volume removed and replaced at each sampling time point using the appropriate mass-balance equation. Three independent dialysis bags were analyzed, and the cumulative release was calculated separately for each bag; the cumulative release percentage was plotted over time. Cumulative release was calculated relative to the measured amount of morin loaded in the nanoparticles.

### 2.5. Characterization of NPs

The hydrodynamic diameter, polydispersity index (PDI), and zeta potential of the lyophilized Morin-PLGA-CS NPs were measured using dynamic light scattering (DLS) with a Malvern Zetasizer Nano ZS (Malvern Panalytical Ltd., Malvern, UK). The lyophilized sample powder was redispersed and sonicated in 10 mM phosphate buffer (pH 7.4) for 5 min before analysis at 25 °C and a backscatter angle of 173°. Three technical measurements were performed on the analyzed dispersion. For scanning electron microscopy (SEM) imaging, a small amount of lyophilized powder was mounted onto double-sided carbon tape on aluminum stubs. The samples were then sputter-coated with a ~10 nm gold–palladium layer using a sputter coater (Quorum Q150R ES, UK) to reduce charging and improve conductivity. The coated samples were examined with a field-emission scanning electron microscope (MIRA3 TESCAN, Czech Republic) at 15 kV and a working distance of 3.88 mm. Images were taken at different magnifications with the InBeam detector to assess surface morphology and apparent dry-particle dimensions. Representative micrographs were collected from multiple fields of view to ensure consistency.

#### Fourier-Transform Infrared (FTIR) Spectroscopy

FTIR spectroscopy was used to interpret the chemical structure and successful incorporation of morin into the PLGA-CS nanoparticle matrix. Spectra of pure morin, blank PLGA-CS NPs, and Morin-PLGA-CS NPs were recorded with a Thermo Scientific Nicolet iS10 spectrometer (Thermo Fisher Scientific, Waltham, MA, USA). For sample preparation, 2 mg of each lyophilized sample was ground with 100 mg of spectroscopic-grade potassium bromide (KBr) to obtain a homogeneous mixture, which was then compressed into transparent discs using a hydraulic press. Spectra were collected in transmission mode over 4000 to 400 cm^−1^ at 4 cm^−1^ resolution and 64 scans per sample. Background spectra were obtained with a pure KBr pellet and automatically subtracted.

### 2.6. Animals and Experimental Design

Adult male Wistar rats (8 weeks old, 200–220 g) were housed under standard laboratory conditions (12 h light/dark cycle, 22 ± 2 °C, 50 ± 10% humidity) with free access to chow and water. Male rats were selected for this initial proof-of-concept study to avoid introducing sex as an additional biological variable in an experiment not designed or powered to assess sex-by-treatment interactions. All procedures were approved by the Institutional Animal Care and Use Committee (IACUC) of Erbil University, Iraq.

Animals were randomly assigned to six treatment groups: (1) non-PTZ vehicle control (0.9% saline with 0.1% DMSO), (2) PTZ-challenged vehicle control, (3) PTZ + free morin (25 mg/kg), (4) PTZ + morin-loaded PLGA-CS NPs (227 mg/kg lyophilized formulation, equivalent to 25 mg/kg morin based on 11% drug loading), (5) PTZ + blank PLGA-CS NPs (227 mg/kg), and (6) PTZ + diazepam (1 mg/kg). Treatments were administered intraperitoneally once daily for 7 consecutive days.

To avoid conceptual overlap between non-convulsive behavioral assays and PTZ-related endpoints, the study was divided into four independent cohorts. Cohort 1 (open-field test (OFT) and elevated-plus maze (EPM); *n* = 8 per group) and Cohort 2 (pentobarbital-induced sleep test; *n* = 8 per group) were non-PTZ cohorts used to assess the intrinsic effects of treatments on locomotor activity and anxiety-like behavior without seizure induction. These cohorts included all treatment groups except the PTZ-challenged vehicle control group, since no PTZ was administered. In contrast, Cohort 3 (PTZ-induced seizure latency, novel object recognition (NOR) test, hippocampal qRT-PCR, and caspase-3 activity at 24 h post-PTZ; *n* = 8 per group) and Cohort 4 (hippocampal and cortical ELISA, hippocampal SOD activity, and ROS measurements at 12 h post-PTZ; *n* = 5 per group) were PTZ-challenged cohorts designed to assess anticonvulsant, cognitive, biochemical, and molecular outcomes after seizure induction. These cohorts included the five PTZ-challenged groups (groups 2–6) plus the non-PTZ vehicle control group (group 1) as a baseline reference. The PTZ-challenged vehicle control group served as the control for seizure-related alterations.

In PTZ-related experiments (Cohorts 3 and 4), PTZ (60 mg/kg, i.p.) was administered 1 h after the final treatment injection on day 7. The 12 h time point captured early post-ictal inflammatory and oxidative changes, whereas the 24 h time point captured later transcriptional and apoptosis-related outcomes; these time points were not intended as definitive peak times for all markers. No animal was used in more than one cohort, and all behavioral, biochemical, and molecular analyses were performed by investigators blinded to treatment allocation. Treatment codes were concealed until data processing was completed.

### 2.7. Behavioral Tests (Anxiolytic and Recognition Memory Effects)

#### 2.7.1. OFT

The OFT assessed spontaneous locomotor activity and anxiety-like behavior in the non-PTZ behavioral Cohort (Cohort 1). On day 7, 1 h after the final treatment injection, rats were placed individually in the center of a square arena (50 × 50 × 50 cm) and allowed to explore for 5 min. Locomotion was recorded with an automated video-tracking system (AnyMaze software (version 7.4, Stoelting Co., Wood Dale, IL, USA)), and the following parameters were quantified: total distance traveled, center-zone distance, center-zone entries, and time spent in the center zone. The arena was cleaned with 70% ethanol between trials [38].

#### 2.7.2. EPM

The EPM was performed 24 h after the OFT in the same non-PTZ Cohort (Cohort 1) to assess anxiety-like behavior. The apparatus had two open arms (50 × 10 cm) and two closed arms (50 × 10 × 20 cm), elevated above the floor, with 50 lux illumination at the maze center. Rats were placed in the central square (10 × 10 cm), facing an open arm, and explored for 5 min. Open- and closed-arm entries and time spent were recorded; an arm entry was defined as all four paws entering the arm. The percentage of open-arm entries was calculated as open-arm entries divided by total arm entries × 100, and open-arm time as open-arm time divided by total arm time × 100. Testing was conducted during the first half of the dark phase, and the maze was cleaned with 70% ethanol after each trial [39].

### 2.8. Evaluation of Hypnotic Potentiation (Pentobarbital-Induced Sleep)

The pentobarbital-induced sleep test was performed in a separate non-PTZ Cohort (Cohort 2) to assess hypnotic potentiation. On day 7, 1 h after the final treatment, sodium pentobarbital (40 mg/kg, i.p.) was administered, and sleep latency and duration were recorded. Sleep latency was the interval from pentobarbital injection to loss of the righting reflex, and sleep duration was the interval from loss to recovery of the righting reflex. All experiments were conducted between 1:00 and 5:00 p.m. [40].

### 2.9. Evaluation of Anticonvulsant Activity (PTZ-Induced Seizure Model)

Anticonvulsant activity was assessed in the PTZ-challenged Cohort (Cohort 3) after the 7-day pretreatment regimen. On day 7, 1 h after the final treatment injection, PTZ (60 mg/kg, i.p.) was administered to induce seizures. Each animal was placed in a transparent Plexiglas cage and observed for 30 min. The onset of the first generalized tonic–clonic seizure was recorded, and seizure latency was defined as the interval from PTZ injection to the first seizure meeting Racine stage 5 criteria [41]. All animals reached the predefined seizure endpoint within the observation period, and no censored observations occurred.

### 2.10. Novel Object Recognition (NOR) Test

To evaluate recognition memory after seizure induction, the NOR test was conducted in the PTZ-challenged Cohort (Cohort 3) after the 7-day drug treatment period. The test was performed in a square arena (50 × 50 × 50 cm) under consistent lighting. On days 5 and 6, animals were habituated to the empty arena for 5 min/day to reduce novelty-induced anxiety. On day 7, immediately after drug administration, animals underwent the familiarization phase, exploring two identical plastic bottle objects (5 cm high) for 5 min. Right after familiarization, all animals received a single intraperitoneal PTZ injection to induce seizures as described in Section 2.9. On day 8, 24 h after familiarization and PTZ administration, the retention test was conducted by replacing one familiar object with a novel object differing in shape and color; each rat explored both objects freely for 5 min (No treatment was administered on day 8). Object exploration was defined as active sniffing or directing the snout within 2 cm of the object; climbing onto, sitting on, or manipulating the object with the paws was not counted as exploratory behavior. The arena and objects were thoroughly cleaned with 70% ethanol between trials to eliminate olfactory cues. Behavioral sessions were recorded using a video-tracking system, and recognition memory was quantified by the discrimination index: (time exploring the novel object—time exploring the familiar object)/total exploration time [42].

### 2.11. ELISA

Hippocampal and cortical tissues were collected from the PTZ-challenged Cohort (Cohort 4) 12 h after PTZ administration (*n* = 5 per group). Animals were transcardially perfused with ice-cold PBS, and brain tissues were rapidly dissected, frozen on dry ice, and stored at −80 °C until analysis. On the day of the assay, tissues were thawed on ice and homogenized in RIPA buffer (1:10 *w*/*v*) containing sodium vanadate and protease inhibitors using a mechanical homogenizer. Homogenates were centrifuged at 14,000× *g* for 15 min at 4 °C, and the supernatants (soluble fraction) were collected. Protein concentration was determined with the Abcam plc (Cambridge, UK). BCA protein assay kit (ab102536) using a bovine serum albumin standard curve. Pro-inflammatory cytokines were measured in the soluble fraction using Rat IL-1β ELISA Kit (Invitrogen, Thermo Fisher Scientific, Waltham, MA, USA; BMS630), Rat IL-6 ELISA Kit (Invitrogen, Thermo Fisher Scientific, Waltham, MA, USA; ERA32RB), and Rat TNF-α ELISA Kit (Abcam plc, Cambridge, UK; ab100785) according to the manufacturers’ instructions. All samples were assayed in duplicate, and cytokine concentrations were interpolated from the standard curves and normalized to total protein content, expressed as pg/mg protein. Blanks and standards were included on each plate.

### 2.12. Quantitative Real-Time PCR (qRT-PCR)

Hippocampal tissues were collected from the PTZ-challenged Cohort (Cohort 3) immediately after the NOR test, about 24 h after PTZ administration (*n* = 8 per group). Total RNA was extracted with TRIzol (Invitrogen), treated with DNase I, and reverse-transcribed into cDNA from 1 µg RNA using a cDNA Reverse Transcription Kit (Applied Biosystems, Thermo Fisher Scientific, Waltham, MA, USA). qRT-PCR was performed on a StepOnePlus™ system with SYBR Green Master Mix (Bio-Rad Laboratories, Hercules, CA, USA) under these cycling conditions: 95 °C for 3 min, followed by 40 cycles of 95 °C for 10 s and 60 °C for 30 s. Primer sequences for Nrf2, HO-1, GFAP, and Iba1 are provided in Table 1. Relative gene expression was calculated by the 2^−ΔΔCt^ method and normalized to GAPDH. All reactions were run in duplicate, and results were expressed relative to the non-PTZ vehicle control group, set to 1.0 and shown as a dashed line in the figures.

### 2.13. SOD Activity Assay

Hippocampal tissues were collected from the PTZ-challenged Cohort (Cohort 4) 12 h after PTZ administration (*n* = 5 per group) to measure SOD activity. Samples were homogenized, centrifuged, and analyzed with a WST-1-based SOD assay kit (ab65354, Abcam) according to the manufacturer’s instructions. Briefly, tissue homogenates were incubated with WST and xanthine oxidase in 96-well plates, and absorbance was measured at 440 nm after incubation at 37 °C. SOD activity was calculated from WST reduction inhibition and expressed as U/mg protein after normalization to total protein measured by the BCA method (Abcam; ab102536). All samples were assayed in duplicate, with reagent blanks included.

### 2.14. Measurement of Oxidative Burden

Hippocampal tissues were collected from the PTZ-challenged Cohort (Cohort 4) 12 h after PTZ administration (*n* = 5 per group) to assess reactive oxygen species. Oxidative burden was measured using a DCFH-DA fluorometric kit (Invitrogen; EEA019), which provides a general index of ROS-related fluorescence. Fresh tissue samples were washed in ice-cold PBS to remove blood and homogenized in nitrogen-purged PBS containing 0.5 mM EDTA under light-protected conditions. DCFH-DA was added and incubated at 37 °C for 30 min in the dark. After centrifugation and washing to remove excess probe, fluorescence was measured at an excitation wavelength of 488 nm and an emission wavelength of 525 nm using a microplate reader. Each assay included a positive control and a probe-free background control, and DCFH-derived fluorescence was normalized to protein content and expressed as relative fluorescence units (RFU)/mg protein.

### 2.15. Caspase-3 Activity Assay (Colorimetric)

Caspase-3 activity was measured in hippocampal tissue from the PTZ-challenged Cohort (Cohort 3), collected immediately after the NOR test, 24 h after PTZ administration (*n* = 8 per group), using a colorimetric assay kit (ab39401, Abcam) according to the manufacturer’s protocol. Tissue samples were homogenized in ice-cold Lysis Buffer IV (50 µL per 5 mg of tissue) and incubated on ice for 10 min. The homogenates were centrifuged at 10,000× *g* for 10 min at 4 °C, and the supernatants were collected. Protein concentration was determined by the BCA assay. For each sample, 50 µg protein was adjusted to 50 µL with Lysis Buffer IV, mixed with 50 µL of 2× Reaction Buffer I containing DTT (10 mM) and 5 µL of DEVD-p-nitroaniline (pNA; 4 mM), and incubated according to the kit instructions. A pNA standard curve was generated using standards ranging from 0 to 200 µM (Supplementary Figure S1), and absorbance was read at 405 nm. Enzyme activity was calculated by linear regression and normalized to total protein, then expressed as fold change relative to the non-PTZ vehicle control group, which was set to 1.0.

### 2.16. Statistical Analysis

IBM SPSS Statistics for Windows, Version 27.0 (IBM Corp., Armonk, NY, USA) was employed for statistical analysis. Data normality was evaluated with the Shapiro–Wilk test, and homogeneity of variance was assessed using Levene’s test. A one-way analysis of variance (ANOVA) for multiple comparisons and Tukey post hoc tests for intergroup comparisons were conducted. Data are shown as mean ± standard deviation (SD), and a *p*-value of <0.05 was considered statistically significant.

## 3. Results

### 3.1. Physicochemical Characterization and Release Profile of Morin-PLGA-CS NPs

**Figure 1:**
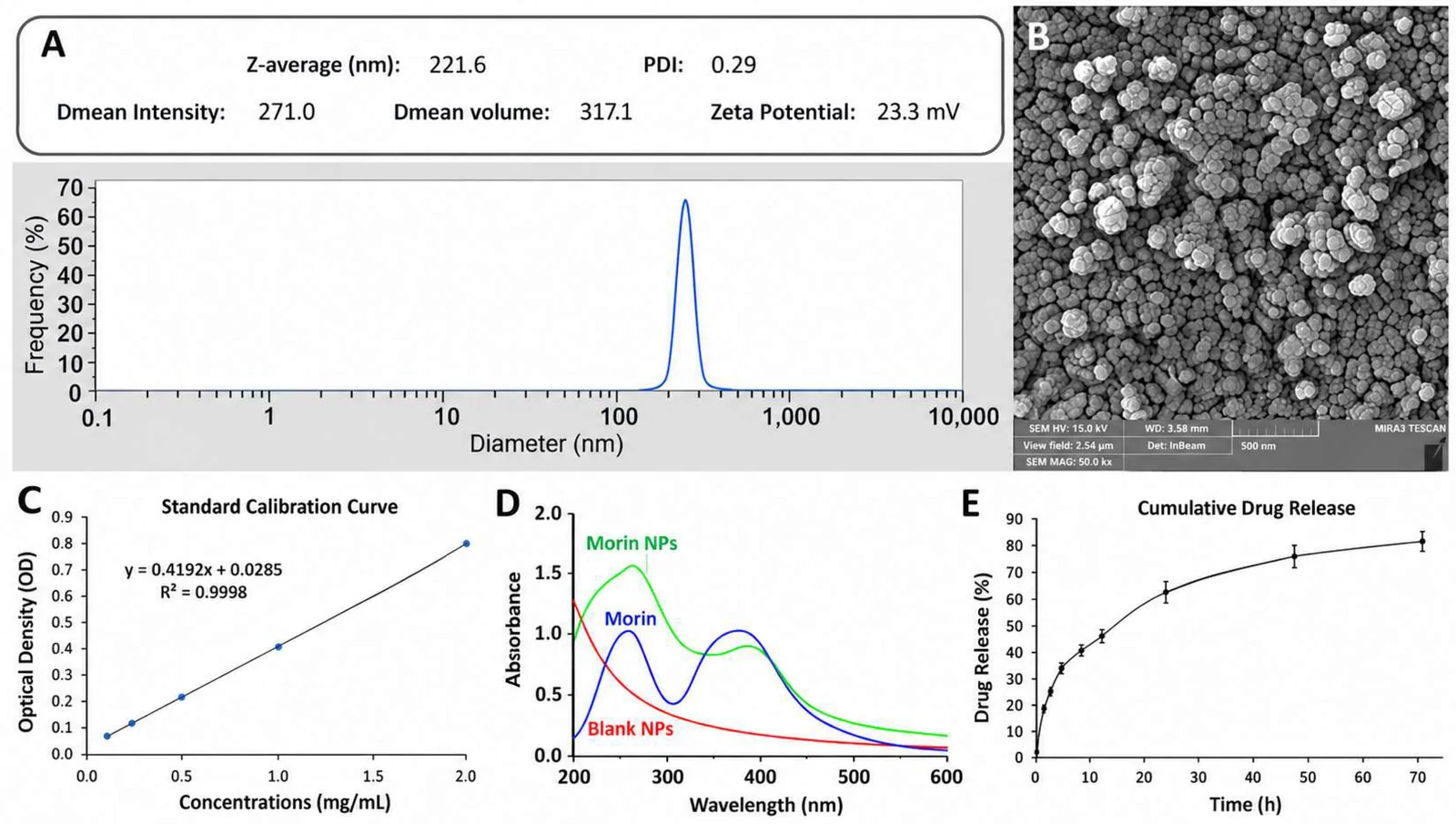
Characterization of Morin-PLGA-CS NPs. (**A**) Dynamic light scattering (DLS) analysis shows a Z-average hydrodynamic diameter of 221.6 nm, a PDI of 0.29, and a zeta potential of +23.3 mV at pH 7.4. (**B**) Scanning electron microscopy (SEM) image depicting spherical and well-dispersed NPs with a relatively uniform size distribution (Scale bar: 500 nm). (**C**) UV-Vis standard calibration curve of morin at 365 nm (linear regression, R^2^ = 0.999). The formulation showed an EE of 81% and a DL of 11%, as determined by the indirect assay described in Section 2.3. (**D**) Full-wavelength UV-Vis absorption spectra (200–600 nm) of free morin, Morin-PLGA-CS NPs, and blank PLGA-CS NPs, supporting the presence of morin in the NPs with a characteristic absorption band around 365 nm. (**E**) Cumulative in vitro morin release profile from PLGA-CS NPs at pH 7.4, showing 34.7% release at 4 h, 75.8% at 48 h, and 81.4% at 72 h.

As shown in Figure 1A, DLS analysis of Morin-PLGA-CS NPs revealed a Z-average hydrodynamic diameter of 221.6 nm (intensity mean: 271.0 nm, volume mean: 317.1 nm) and a PDI of 0.29. The zeta potential was +23.3 mV at pH 7.4, suggesting acceptable colloidal stability under the tested conditions. The morphology was further confirmed by SEM; the NPs were predominantly spherical and well-dispersed, with particle sizes mainly ranging from 150 to 250 nm and no obvious aggregation (Figure 1B and Figure S2).

According to the standard calibration curve of morin (Figure 1C), the formulation achieved an EE of 81% and a drug-loading content of 11%, reflecting efficient incorporation of morin within the PLGA-CS matrix. Based on the 11% drug loading, a dose of 227 mg/kg of lyophilized Morin-PLGA-CS NPs was calculated to deliver an equivalent morin dose of 25 mg/kg, matching the free-morin group for valid comparison. As shown in Figure 1D, the full-wavelength UV–Vis spectra showed morin-associated absorption in the Morin-PLGA-CS NP dispersion, consistent with the presence of morin in the formulation. Free morin showed the characteristic flavonoid absorption profile, with a major peak near 260 nm and a broad shoulder around 380 nm. The blank NPs showed a decreasing scattering background with no distinct peaks. Additionally, the loaded NPs displayed morin-related absorption superimposed on the polymer baseline, notably an enhanced band around 365 nm (Figure 1D). The in vitro release profile exhibited a biphasic pattern (Figure 1E), with an initial burst release of 34.7% in the first 4 h, followed by a release phase reaching 75.8% at 48 h and approximately 81.4% at 72 h.

#### FTIR

FTIR spectroscopy was used to assess chemical interactions and confirm the successful incorporation of morin into the PLGA–CS nanoparticle system (Table 2). Free morin showed characteristic bands at 3405 cm^−1^ (O–H stretching), 1661 cm^−1^ (conjugated C=O stretching), 1308 cm^−1^ (C–OH-related vibration of morin), and 1253 cm^−1^ (C–O–C stretching), consistent with previously reported spectra. The blank PLGA-CS NPs exhibited the PLGA ester carbonyl band at 1750 cm^−1^ and the CS amide I and II bands at 1648 and 1588 cm^−1^, consistent with the presence of PLGA- and chitosan-associated functional groups. After morin loading, the conjugated C=O band shifted from 1661 to 1645 cm^−1^ and overlapped with CS’s amide I band, consistent with noncovalent interactions, likely hydrogen bonding, between morin and the polymer matrix. The 1308 cm^−1^ band decreased markedly, and the 1253 cm^−1^ band became much weaker, suggesting possible interactions involving morin hydroxyl and ether groups in intermolecular interactions. The aromatic C–H out-of-plane vibration at 831 cm^−1^ also became very weak or nearly undetectable after encapsulation, further supporting incorporation of morin into the nanoparticle system. Importantly, the PLGA ester carbonyl band at 1750 cm^−1^ showed only a slight decrease in intensity and remained essentially unchanged at 1749 cm^−1^, indicating preservation of the polymer backbone. The characteristic CS amide bands were also retained with only minor shifts, indicating retention of CS-associated functional groups after morin loading. Overall, these spectral changes suggest successful encapsulation of morin within the PLGA–CS NPs via hydrogen bonding and other weak intermolecular interactions.

### 3.2. Behavioral Outcomes in the OFT

**Figure 2:**
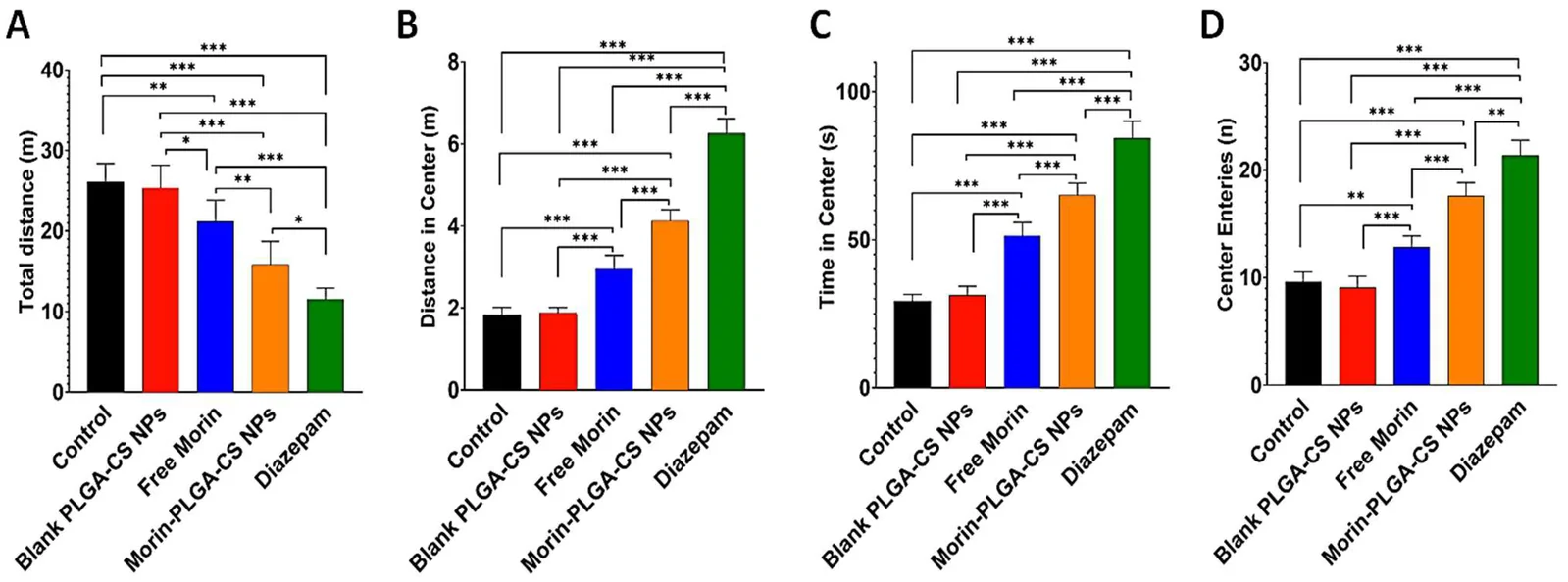
Evaluation of locomotor activity and anxiety-related behavior using the Open-Field Test (OFT). (**A**) Total distance traveled, showing a significant reduction in the Morin-PLGA-CS NPs and Diazepam groups compared to the vehicle control group. (**B**) Distance traveled in the center, (**C**) center-zone entries, and (**D**) center-zone time, all demonstrating a significant increase in center-zone exploration despite reduced total distance traveled for the Morin-PLGA-CS NPs and Diazepam groups relative to the vehicle control and free-morin groups. Diazepam exhibited the most potent anxiolytic effect. Data are shown as mean ± SD (*n* = 8 per group). * *p* < 0.05, ** *p* < 0.01, and *** *p* < 0.001.

The OFT was used to assess locomotor activity and anxiety-like behavior. Blank PLGA-CS NPs did not differ significantly from the vehicle control group, indicating that the carrier itself had no detectable intrinsic behavioral effect. In contrast, treatment with Morin-PLGA-CS NPs and diazepam significantly reduced the total distance traveled compared with the vehicle control (Figure 2A; *p* < 0.01), indicating a reduction in locomotor activity. At the same time, Morin-PLGA-CS NPs significantly increased the distance traveled in the center zone (Figure 2B), the number of center entries (Figure 2C), and the time spent in the center (Figure 2D) compared with the vehicle control group, free morin, and blank NP groups (*p* < 0.01). Diazepam produced the largest increase in center-zone exploration time and entry frequency (*p* < 0.01).

### 3.3. Anxiolytic Profile in the EPM

**Figure 3:**
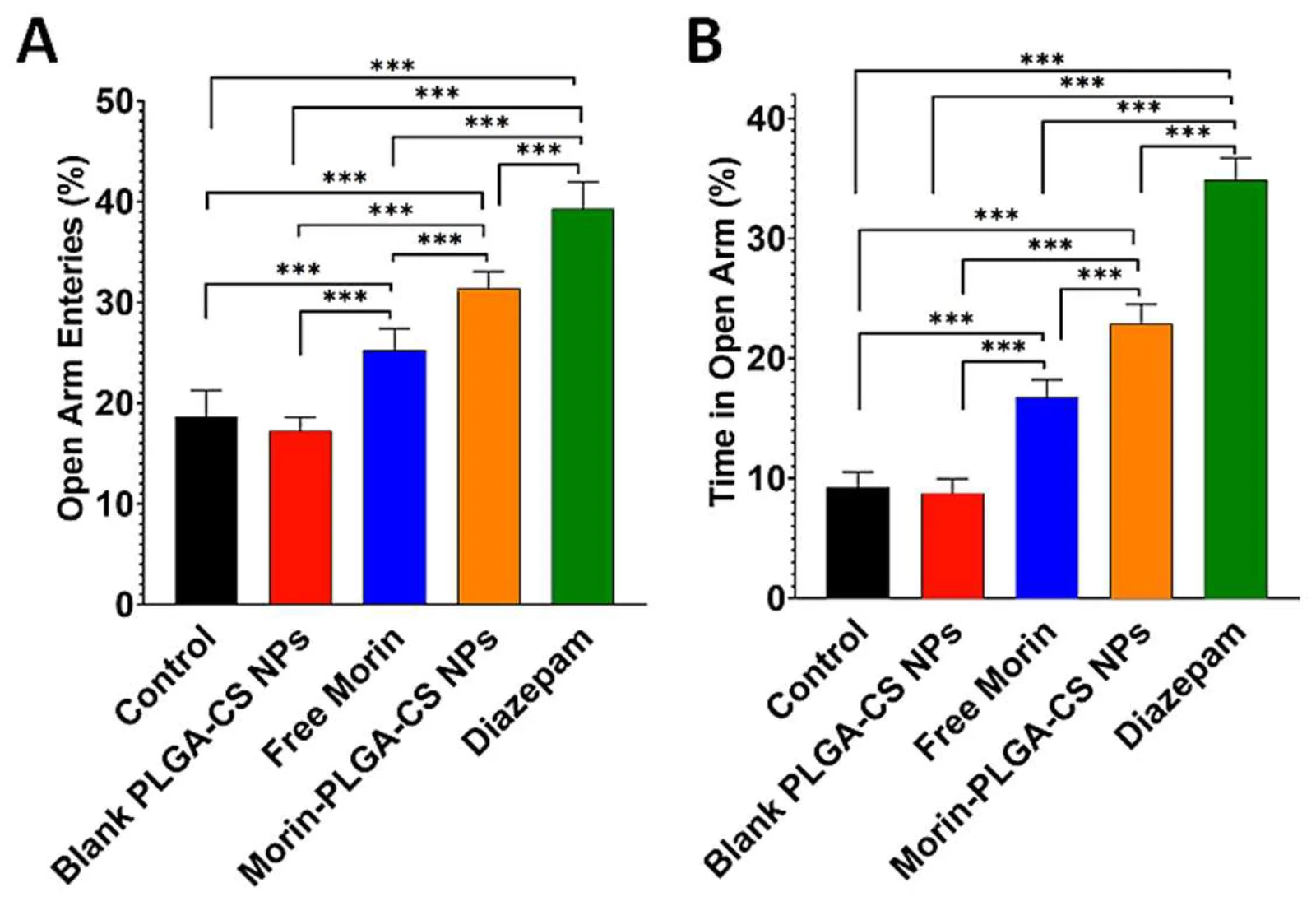
Assessment of anxiolytic-like behavior using the Elevated-Plus Maze (EPM) test in the non-PTZ Cohort (Cohort 1). (**A**) Percentage of open-arm entries and (**B**) percentage of time spent in the open arms. The Diazepam group showed the highest values (39.3% and 34.9%, respectively), while the Morin-PLGA-CS NP group (31.4% and 22.9%) demonstrated significantly enhanced anxiolytic activity compared to the free-morin group (25.3% and 16.8%). The blank PLGA-CS NP group showed behavior comparable to the vehicle control group. Data are shown as mean ± SD (*n* = 8 per group). *** *p* < 0.001.

In the EPM test (Figure 3), diazepam-treated rats showed a significant increase in the percentage of open-arm entries (39.3%) and time spent in the open arms (34.9%) compared with the vehicle control group (18.7% and 9.5%, respectively; *p* < 0.001). Morin-PLGA-CS NPs also improved anxiety-like behavior, increasing open-arm entries to 31.4% and time spent in open arms to 22.9%, compared with the free-morin group (25.3% and 16.8%, respectively; *p* < 0.001). In contrast, the blank PLGA-CS NP group behaved similarly to the vehicle control group, confirming the absence of an inherent anxiolytic effect attributable to the nanocarrier alone.

### 3.4. Pentobarbital-Induced Sleep Test

**Figure 4:**
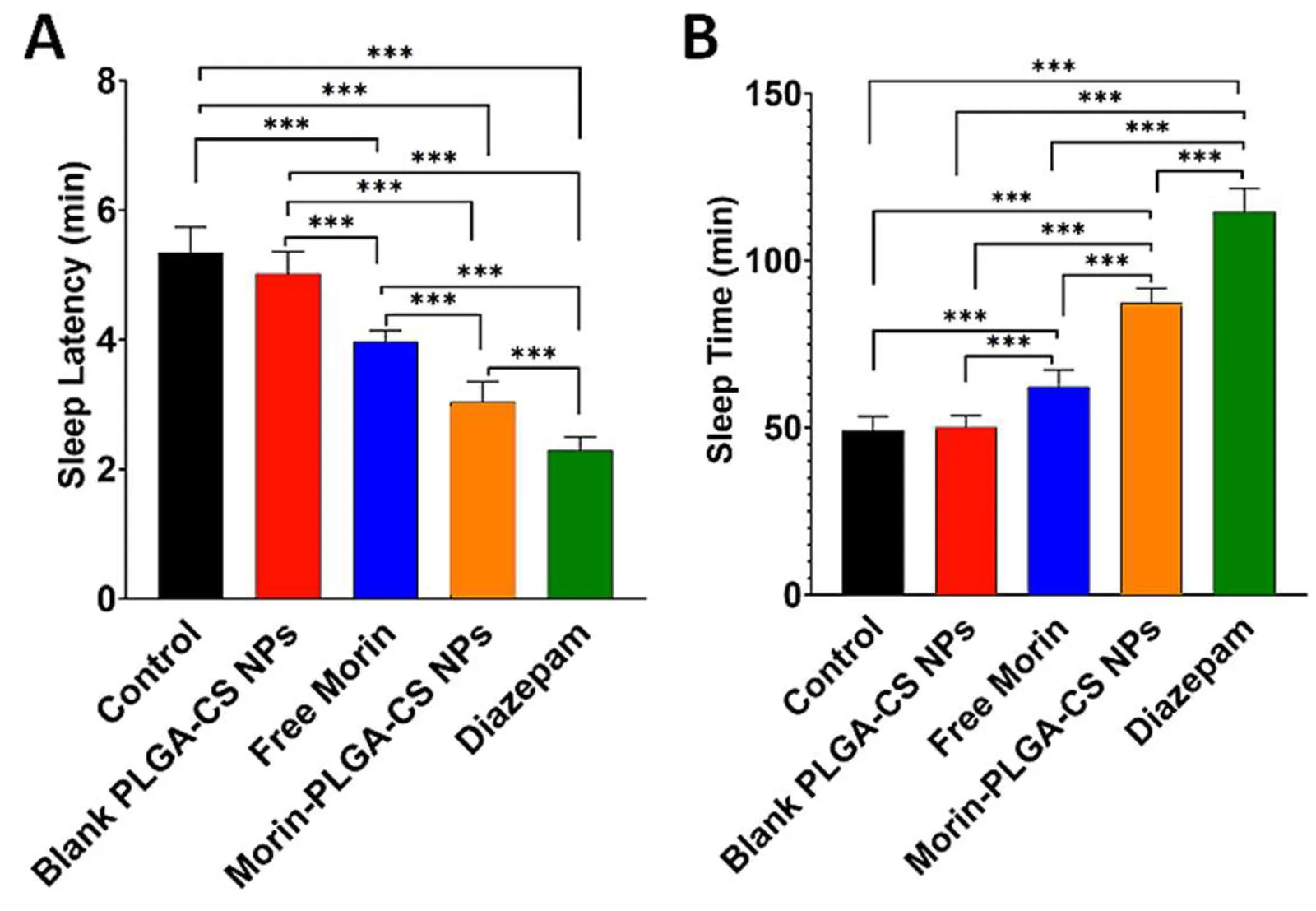
Evaluation of sedative potential using the pentobarbital-induced sleep test in the non-PTZ Cohort (Cohort 2). (**A**) Sleep latency and (**B**) sleep duration. The Morin-PLGA-CS NP group exhibited a significantly reduced sleep latency (3.0 min) and prolonged sleep duration (87.5 min) compared to the free-morin group (3.97 min and 62.3 min, respectively), indicating enhanced hypnotic activity. Data are shown as mean ± SD (*n* = 8 per group). *** *p* < 0.001.

The pentobarbital-induced sleep test (Figure 4) showed that the vehicle control group had a mean sleep latency of 5.4 min and a sleep duration of 49.3 min. Diazepam markedly reduced sleep latency to 2.3 min and increased sleep duration to 114.6 min (*p* < 0.001). The blank PLGA-CS NP group showed the longest latency (5.0 min) and the shortest duration (50.1 min), closely resembling the control group. Free morin reduced sleep latency to 3.97 min and increased sleep duration to 62.3 min. In contrast, Morin-PLGA-CS NPs produced a stronger sedative effect, shortening sleep latency to 3.0 min and prolonging sleep duration to 87.5 min compared with free morin (*p* < 0.001). Although less potent than diazepam, the nanoparticle formulation clearly enhanced the hypnotic activity of morin.

### 3.5. Anticonvulsant Activity in the PTZ Model

**Figure 5:**
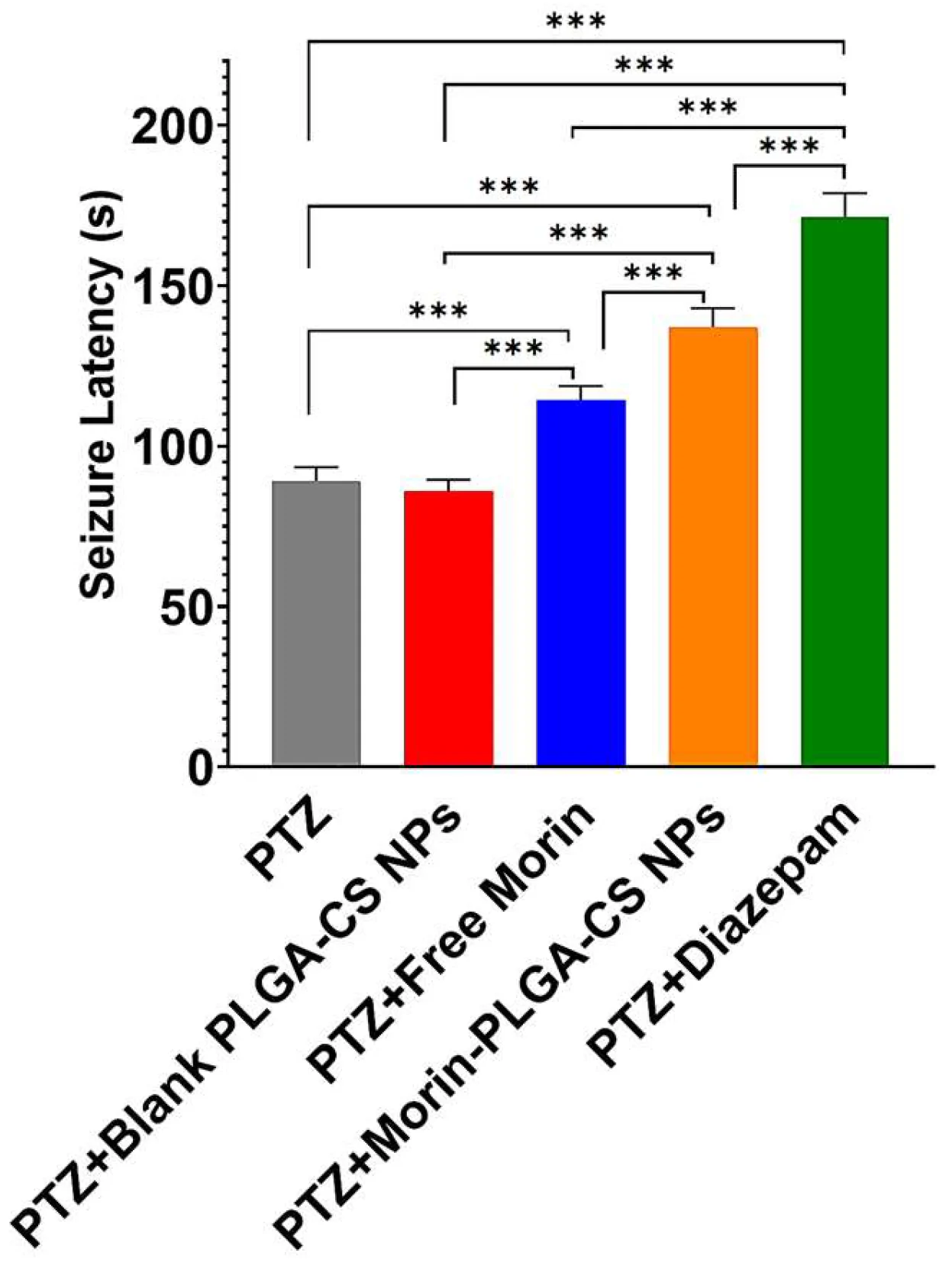
Anticonvulsant activity assessed by seizure latency following PTZ administration in the PTZ-challenged Cohort (Cohort 3). Latency was 88.9 s in the PTZ-challenged vehicle control, 114.5 s after free morin, 137.2 s after Morin-PLGA-CS NPs, and 171.5 s after diazepam. Data are shown as mean ± SD (*n* = 8 per group). *** *p* < 0.001.

All animals reached the predefined endpoint within the observation period. As shown in Figure 5, PTZ administration induced seizures with an onset latency of 88.9 s in the PTZ-challenged vehicle control. Treatment with Morin-PLGA-CS NPs significantly prolonged seizure latency to 137.2 s. This effect was significantly greater than that of free morin, which prolonged latency to 114.5 s (*p* < 0.001). Diazepam exerted the strongest anticonvulsant action, extending seizure latency to 171.5 s (*p* < 0.001). These data indicate that nanoencapsulation significantly improved the anticonvulsant efficacy of morin.

### 3.6. Recognition Memory in the NOR Test

**Figure 6:**
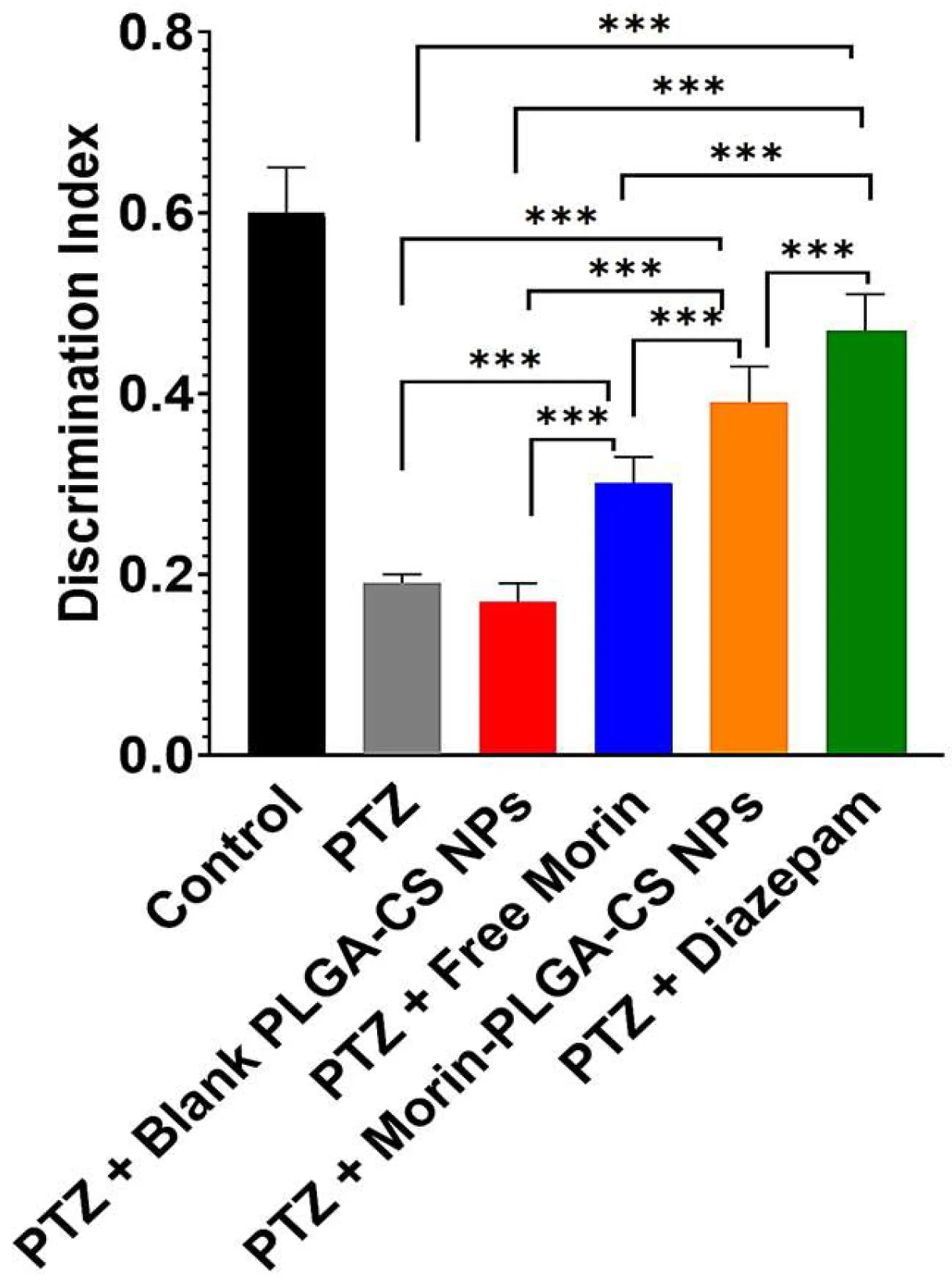
Recognition memory assessed by the Novel Object Recognition (NOR) test in the PTZ-challenged Cohort (Cohort 3). The discrimination index (DI) was significantly reduced in the PTZ-challenged vehicle group (0.19) compared to the non-PTZ vehicle control group (0.6), indicating cognitive impairment. Treatment with Morin-PLGA-CS NPs resulted in a higher DI (0.39) than free morin (0.3), demonstrating improved recognition memory performance under the experimental conditions. Data are shown as mean ± SD (*n* = 8 per group). *** *p* < 0.001.

Recognition memory was assessed using the NOR test (Figure 6). The non-PTZ vehicle control showed a DI of 0.60. In contrast, PTZ administration reduced the DI to 0.19, indicating marked recognition memory impairment (*p* < 0.001). Morin-PLGA-CS NPs improved performance, increasing the DI to 0.39 compared with 0.30 in the free-morin group, indicating a superior ability of the nanoparticle formulation to alleviate recognition memory deficits (*p* < 0.001). Diazepam was associated with a higher DI (0.47) under the tested conditions.

### 3.7. Neuroinflammatory Cytokines in the Cortex and Hippocampus

**Figure 7:**
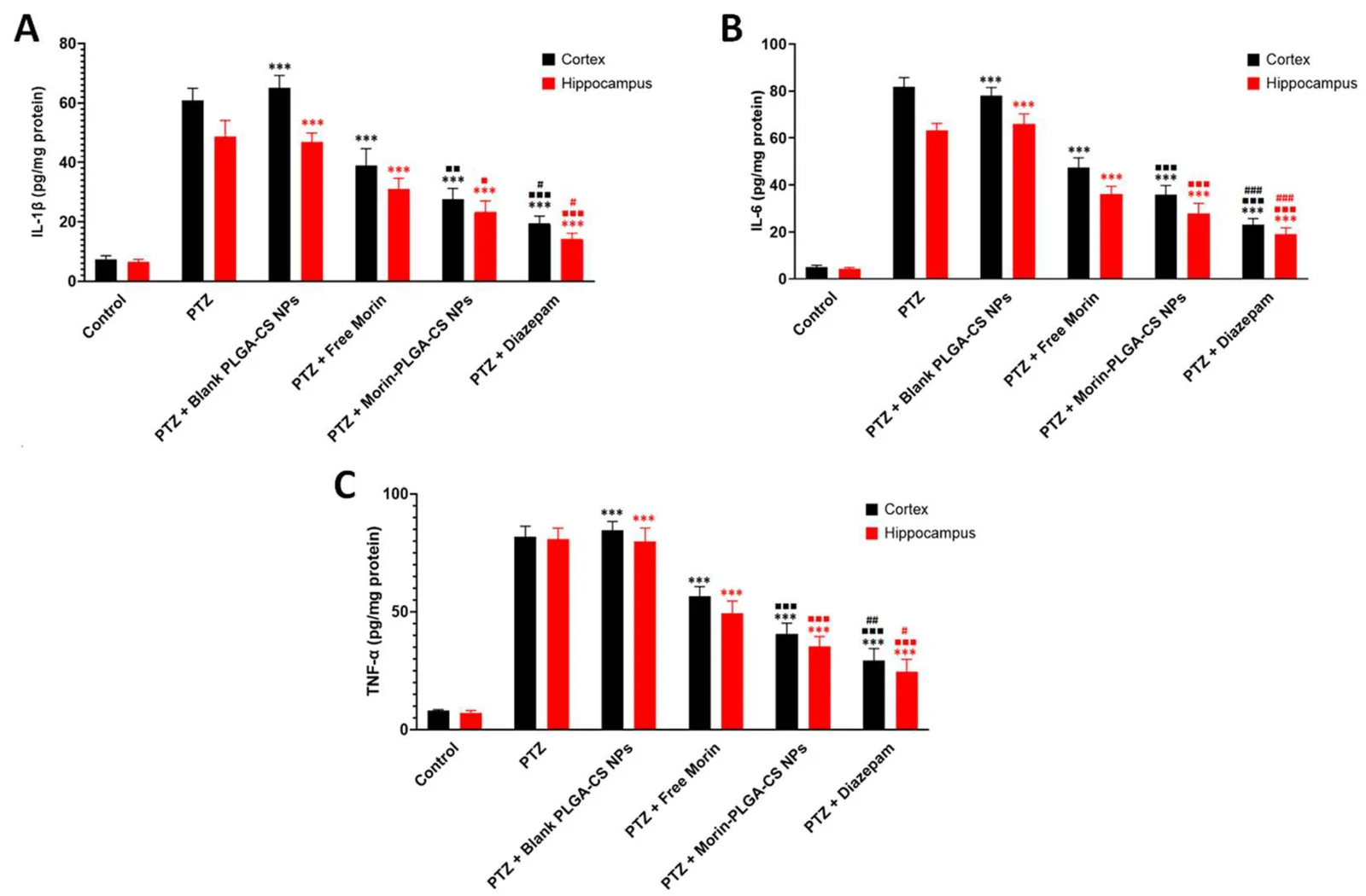
Effects of treatments on pro-inflammatory cytokine levels in the cortex and hippocampus measured by ELISA. Levels of (**A**) IL-1β, (**B**) IL-6, and (**C**) TNF-α were significantly elevated 12 h following PTZ administration. Morin-PLGA-CS NP treatment effectively attenuated this neuroinflammatory response, significantly reducing all three cytokine levels in both brain regions compared to the PTZ group. Data are shown as mean ± SD (*n* = 5 per group). Statistical significance was set at (*p* < 0.05). Significant differences are indicated as follows: *** *p* < 0.001 versus PTZ control; ■/■■/■■■, *p* < 0.05/*p* < 0.01/*p* < 0.001 versus PTZ + free morin; and #/##/###, *p* < 0.05/*p* < 0.01/*p* < 0.001 versus PTZ + Morin-PLGA-CS NPs. Comparisons of cortical tissue data are depicted in black, whereas those of hippocampal tissue data are depicted in red.

To clarify the anti-inflammatory mechanism, IL-1β, IL-6, and TNF-α levels were measured in both the cortex and hippocampus (Figure 7A–C). PTZ administration markedly increased cytokine levels in both regions. For IL-1β, cortical levels rose from 7.28 pg/mg in the non-PTZ vehicle-treated control to 60.94 pg/mg in the PTZ-challenged vehicle control (*p* < 0.001). Morin-PLGA-CS NPs reduced cortical IL-1β to 27.56 pg/mg and hippocampal IL-1β to 23.4 pg/mg, compared with 48.7 pg/mg in the PTZ-challenged vehicle control (*p* < 0.001). For IL-6, cortical levels increased to 81.64 pg/mg after PTZ induction and were reduced to 36.28 pg/mg by Morin-PLGA-CS NPs. In the hippocampus, IL-6 declined from 62.18 pg/mg in the PTZ-challenged vehicle control to 26.6 pg/mg (*p* < 0.001). For TNF-α, cortical levels increased from 8.12 pg/mg in non-PTZ vehicle-treated control to 81.72 pg/mg after PTZ exposure, and Morin-PLGA-CS NPs reduced this value to 40.7 pg/mg (*p* < 0.001). In the hippocampus, TNF-α decreased from 80.82 pg/mg in the PTZ-challenged vehicle control to 35.32 pg/mg after Morin-PLGA-CS NP treatment (*p* < 0.001).

### 3.8. Oxidative Stress, Apoptosis, and Glial Markers

**Figure 8:**
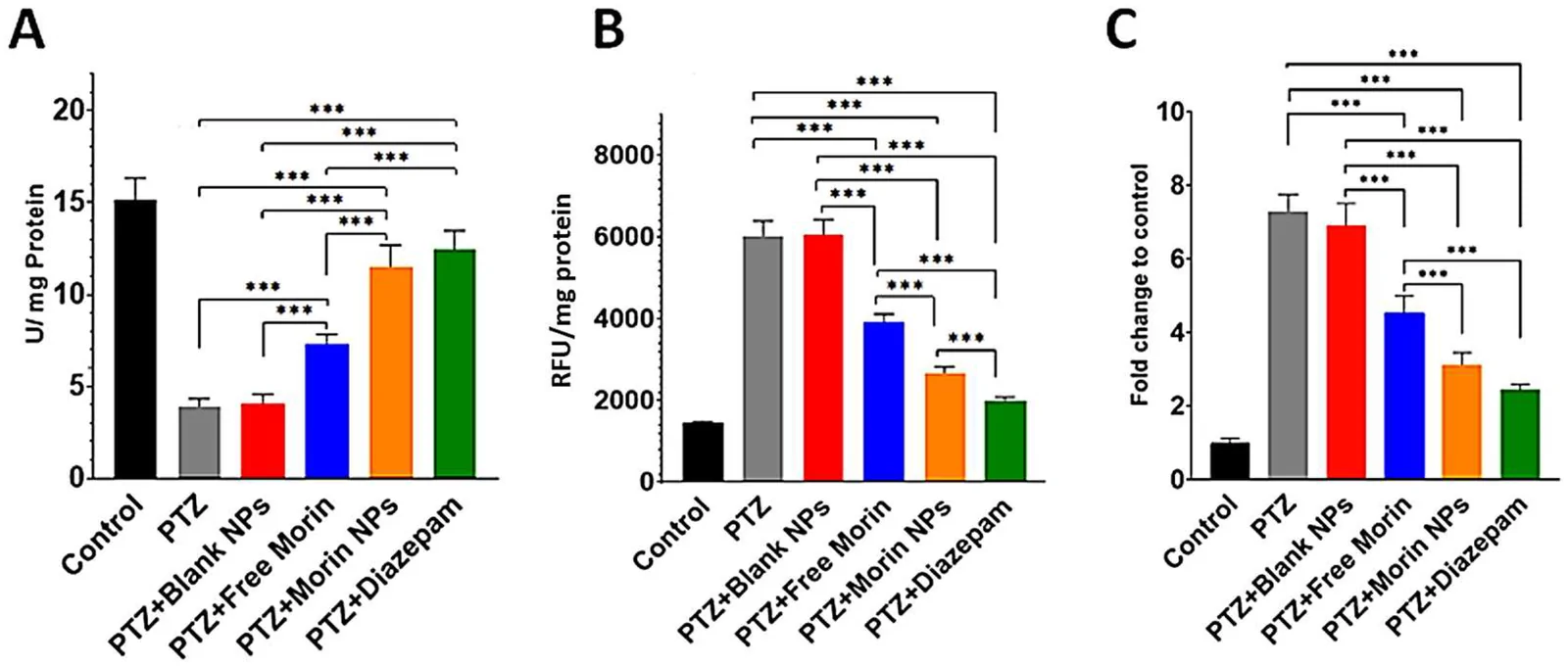
Biochemical evaluation of oxidative stress and apoptosis in hippocampal tissue post-treatment. (**A**) SOD activity (U/mg protein) indicated antioxidant defense; PTZ significantly reduced SOD activity, while Morin-PLGA-CS NPs restored it more effectively than free morin (Cohort 4, 12 h post-PTZ, *n* = 5). (**B**) DCF fluorescence (RFU/mg protein) showed general oxidative burden. PTZ increased ROS production, which was mitigated by treatments, particularly with Morin-PLGA-CS NPs (Cohort 4, 12 h post-PTZ, *n* = 5). (**C**) Caspase-3 activity expressed as fold change relative to the non-PTZ vehicle control group (Cohort 3, 24 h post-PTZ, *n* = 8). PTZ heightened caspase-3 activation, but treatments decreased apoptosis, with NPs having a greater impact than free morin. Data are shown as mean ± SD. *** *p* < 0.001.

As shown in Figure 8A, PTZ significantly reduced hippocampal SOD activity to 3.88 ± 0.44 U/mg protein compared with 15.14 ± 1.19 U/mg protein in the non-PTZ vehicle-treated control (*p* < 0.001), indicating impaired antioxidant defense. Blank NPs did not meaningfully restore SOD activity (4.02 ± 0.54 U/mg protein), whereas free morin significantly increased SOD activity to 7.34 ± 0.48 U/mg protein (*p* < 0.001). Morin-PLGA-CS NPs produced a greater recovery, raising SOD activity to 11.54 ± 1.17 U/mg protein, although this remained significantly lower than the non-PTZ vehicle-treated control level (*p* < 0.001). Diazepam also increased SOD activity to 12.44 ± 1.04 U/mg protein relative to PTZ (*p* < 0.001), approaching but remaining below the non-PTZ vehicle-treated control value.

DCF fluorescence, as a general oxidative-burden index in hippocampal tissue, expressed as RFU/mg protein, is shown in Figure 8B. PTZ increased ROS to 6020 ± 384 RFU/mg protein compared with 1446 RFU/mg protein in the non-PTZ vehicle-treated control (*p* < 0.001). Blank NPs did not significantly alter PTZ-induced DCF fluorescence (6078 ± 355 RFU/mg protein). Free morin reduced DCF fluorescence to 3938 ± 185 RFU/mg protein (*p* < 0.001), whereas Morin-PLGA-CS NPs produced a more pronounced reduction to 2689 ± 141 RFU/mg protein (*p* < 0.001). Diazepam decreased DCFH-derived fluorescence to 1997 ± 93 RFU/mg protein, showing a significantly stronger redox-associated effect than the other treatment groups (*p* < 0.001).

Caspase-3 activity, expressed as fold change relative to the non-PTZ vehicle-treated control group, is shown in Figure 8C. PTZ markedly increased caspase-3 activity by 7.3-fold (7.27 ± 0.51) versus the non-PTZ vehicle-treated control (*p* < 0.001). Blank NPs did not significantly alter this response compared with the PTZ group. Free morin partially reduced the PTZ-induced increase to 4.54 ± 0.45 (*p* < 0.001), whereas Morin-PLGA-CS NPs produced a greater reduction to 3.12 ± 0.33 (*p* < 0.001). Diazepam showed the lowest fold change (2.43 ± 0.16); however, this decrease was not statistically significant compared with the Morin-PLGA-CS NP group.

### 3.9. Hippocampal qRT-PCR Analysis of Antioxidant and Glial Genes

**Figure 9:**
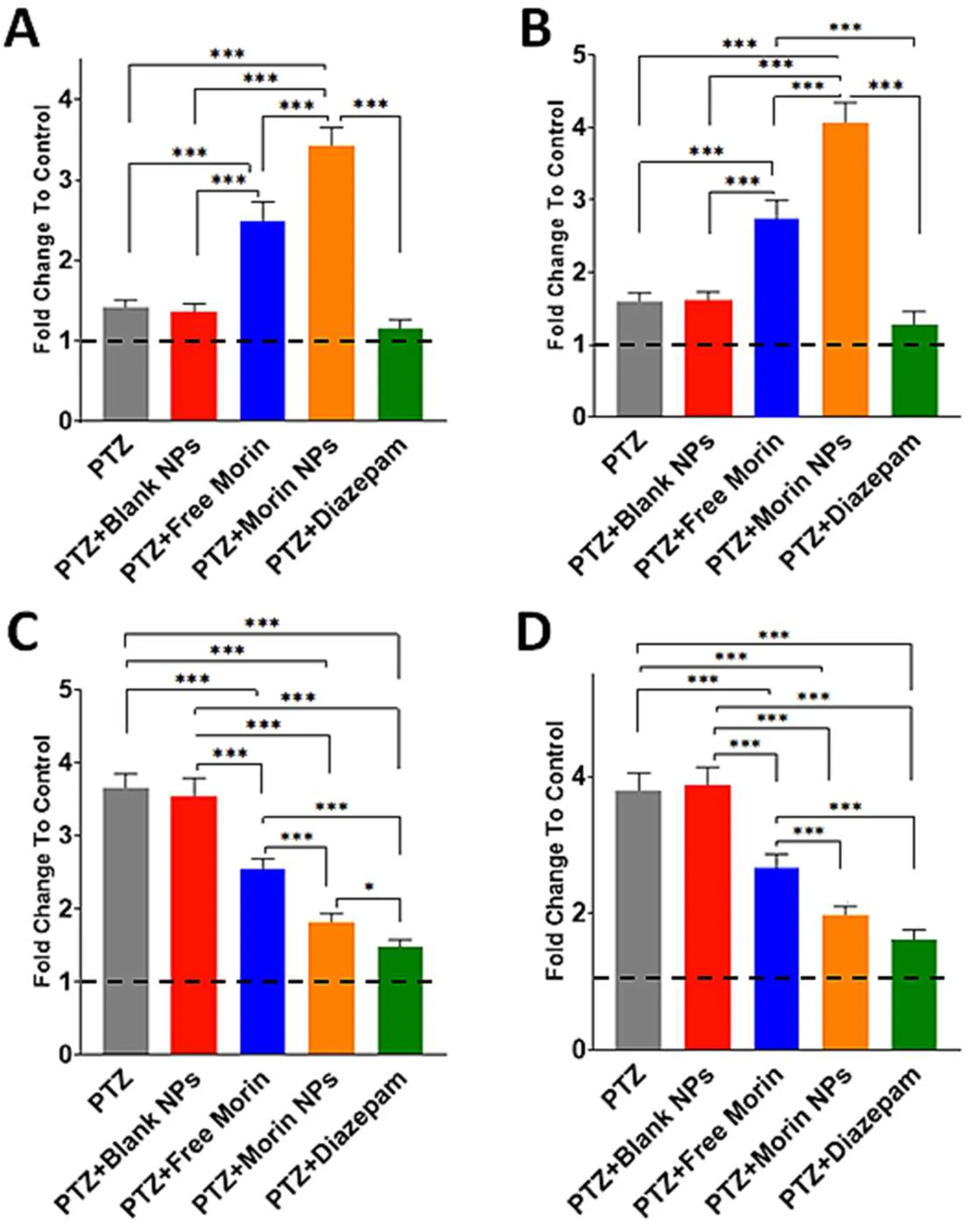
Hippocampal qRT-PCR analysis of Nrf2, HO-1, GFAP, and Iba1 transcript expression following PTZ-induced seizures. Effects of free morin, Morin-PLGA-CS NPs, and diazepam on hippocampal expression of Nrf2 (**A**), HO-1 (**B**), GFAP (**C**), and Iba1 (**D**) in PTZ-induced seizure rats. Data are presented as mean ± SD and expressed as fold change relative to the non-PTZ vehicle-treated control group (set to 1.0, indicated by the dashed line). Samples were collected 24 h after PTZ administration. Data are shown as mean ± SD (*n* = 8 per group). * *p* < 0.05 *** *p* < 0.001.

As shown in Figure 9, PTZ administration significantly increased hippocampal GFAP mRNA expression to 3.65 ± 0.20 and Iba1 mRNA expression to 3.80 ± 0.25 relative to the non-PTZ vehicle-treated control. These transcript-level changes are consistent with, but not definitively confirmatory of, reactive astrogliosis and microglial activation. At the same time, Nrf2 and HO-1 were modestly increased in the PTZ-challenged vehicle control (1.41 ± 0.10 and 1.59 ± 0.12, respectively), consistent with a compensatory antioxidant response. Blank PLGA-CS NPs did not meaningfully alter these transcript levels compared with the PTZ-challenged vehicle control (Nrf2: 1.37 ± 0.09; HO-1: 1.61 ± 0.12; GFAP: 3.53 ± 0.26; Iba1: 3.88 ± 0.26).

Free morin increased Nrf2 to 2.48 ± 0.24 and HO-1 to 2.74 ± 0.25, while reducing GFAP to 2.54 ± 0.14 and Iba1 to 2.66 ± 0.20. Morin-PLGA-CS NPs produced a significantly stronger effect, further elevating Nrf2 to 3.42 ± 0.23 and HO-1 to 4.07 ± 0.27, while reducing GFAP and Iba1 to 1.82 ± 0.11 and 1.98 ± 0.12, respectively (all *p* < 0.001 vs. free morin and PTZ groups). In contrast, diazepam restored Nrf2 and HO-1 to near-control values (1.16 ± 0.10 and 1.29 ± 0.17, respectively) and partially suppressed GFAP (1.48 ± 0.09) and Iba1 (1.63 ± 0.13).

## 4. Discussion

Despite considerable advances in antiseizure pharmacotherapy over the past three decades, the clinical landscape of epilepsy management remains characterized by a persistent therapeutic ceiling [2]. Approximately one-third of patients continue to experience breakthrough seizures, while those who achieve seizure control often face dose-limiting cognitive impairment, sedation, and neuropsychiatric comorbidities [43]. This dilemma arises partly because most conventional ASMs were optimized for receptor-binding potency rather than favorable biodistribution, metabolic stability, or selective CNS targeting [44]. Natural flavonoids like morin have been recognized for their multi-target neuroprotective potential, including GABAergic modulation, antioxidant defense, and anti-inflammatory signaling [45,46,47]. However, their clinical translation has been limited by poor aqueous solubility, extensive first-pass metabolism, and poorly characterized brain exposure. The present study aimed to improve the therapeutic efficacy of morin through a nanoencapsulation strategy by encapsulating it within a PLGA-CS polymeric matrix. By integrating behavioral, cognitive, and anticonvulsant endpoints in a PTZ-challenged rodent model, this investigation provides an assessment of a nanotherapeutic platform.

The study showed that nanoencapsulation of morin within PLGA-CS NPs produced greater effects than free morin across several measured endpoints under the present experimental conditions: sedation, anxiolysis, memory preservation, and neuroinflammatory suppression. Morin-PLGA-CS NPs increased seizure latency to 137.2 s, representing an approximately 54% improvement over the PTZ-challenged vehicle control (88.9 s) and an approximately 20% improvement over free morin (114.5 s). However, because seizure severity and duration were not comprehensively analyzed, the anticonvulsant effect is limited to prolongation of latency to the predefined seizure endpoint. Importantly, the 227 mg/kg dose of lyophilized Morin-PLGA-CS NPs contained 25 mg/kg of morin (based on 11% drug loading), ensuring the nanoparticle group received the same active morin dose as the free-morin group and allowing direct comparison of the encapsulation strategy. In the pentobarbital-induced sleep test, this formulation reduced sleep latency by 43% from 5.4 min in the vehicle group to 3.0 min in the Morin-PLGA-CS NP group. Further, sleep duration increased from 49.3 min in the vehicle group to 87.5 min in the Morin-PLGA-CS NP group and was approximately 40% higher than in the free-morin group (62.3 min). The blank PLGA-CS NP group showed similar seizure latency and sleep parameters as the PTZ-challenged vehicle control, confirming its pharmacological inertness. Anxiolytic assessments revealed that Morin-PLGA-CS NP significantly increased center distance and time in the open-field test and open-arm entries in the elevated-plus maze, indicating enhanced anxiolytic effects. The blank PLGA-CS NP group showed no intrinsic anxiolytic or anxiogenic effects.

A noteworthy observation arises when examining the anxiolytic effects of Morin-PLGA-CS NPs. In the OFT, this NP formulation reduced total distance traveled by 39% and increased center exploration time by over 240% relative to the vehicle control group. While Morin-PLGA-CS NPs increased center-zone exploration, the concurrent reduction in total distance traveled indicates that motor slowing, reduced exploratory drive, or sedation may have contributed to the behavioral profile. This prevents a definitive interpretation of these findings as a clear pharmacological separation between anxiolysis and sedation. Diazepam exhibited a similar pattern but with greater effects in both domains, consistent with its potent modulation of gamma-aminobutyric acid type A (GABA_A_) receptors [48]. In contrast, free morin had modest effects [49]. The NOR test data showed that Morin-PLGA-CS NPs achieved a DI of 0.39, marking a 105% improvement over the PTZ-challenged vehicle group (0.19) and a 30% improvement over free morin (0.30). Improved NOR performance under the tested conditions is notable, and the Morin-PLGA-CS NPs provided significantly better protection against PTZ-induced memory deficits. The cognitive findings correlate with the ELISA results, indicating that PTZ administration significantly increased pro-inflammatory cytokines IL-1β, IL-6, and TNF-α, which are known to create a neuroinflammatory environment that has been associated with memory impairment in experimental models [50]. Compared to free morin, Morin-PLGA-CS NPs reduced cytokine levels more than the PTZ-challenged vehicle control. It must be noted that tissues for ELISA and oxidative stress assays were collected 12 h after PTZ administration to assess the early subacute phase of seizure-induced neuroinflammatory and oxidative changes. The 12 h time point was selected to assess an early post-ictal interval; it was not assumed to represent the peak response for all markers.

In addition, the molecular analyses revealed a key distinction between Morin-PLGA-CS NPs and diazepam, particularly in relation to the Nrf2/HO-1 mRNA expression. In the acute PTZ model, seizure induction causes oxidative stress and neuroinflammation, prompting an upregulation of the Nrf2/HO-1 axis as a defensive response [51,52]. This might explain the PTZ group’s slightly elevated expression of these genes, accompanied by higher ROS levels and reduced antioxidant capacity. Diazepam, by modulating GABA_A_ receptors, reduces neuronal hyperexcitability and reduces seizure activity, decreasing the need for endogenous antioxidant compensation [53,54]. This may explain normalized Nrf2/HO-1 expression, lower glial activation, and reduced apoptotic signaling [55]. In contrast, Morin-PLGA-CS NPs had weaker anticonvulsant effects but reduced oxidative and inflammatory markers, suggesting benefits beyond seizure suppression. Previous studies suggest that morin may activate Nrf2 signaling via kinases such as PI3K/Akt and MAPKs, enhancing transcription of antioxidant enzymes [36]. This redox-modulating effect corresponds with reduced DCF fluorescence, interpreted as a decrease in general oxidative-burden levels, restored SOD activity, and suppressed Caspase-3, indicating decreased oxidative stress-induced apoptosis. Thus, while diazepam indirectly acts by eliminating oxidative injury triggers, the findings suggest an association between Morin-PLGA-CS NP treatment and enhanced redox-related responses. These findings suggest Morin-PLGA-CS NPs may provide a complementary neuroprotective approach by reducing oxidative stress and apoptotic signaling after acute seizures. Whether these acute changes translate into long-term benefits remains unknown and requires testing in chronic epilepsy models. It must be mentioned that, beyond the classical enzymatic antioxidant systems, the brain also relies on nonenzymatic redox regulators such as ascorbate, which is abundant in neural tissue and plays an important role in maintaining oxidative balance [56]. In seizure models, ascorbate has been linked to the attenuation of oxidative damage, reduced lipid and protein oxidation, and the modulation of seizure-related neurotoxicity. This indicates that ascorbate contributes to the broader endogenous defense network during epileptic stress [57]. Within this framework, the protective profile observed with morin-loaded NPs can be interpreted as part of a multifaceted antioxidant and anti-inflammatory response that reinforces intrinsic redox homeostasis rather than acting through a single isolated mechanism.

According to the qRT-PCR data, seizure induction by PTZ produced a modest but significant upregulation of hippocampal Nrf2 and HO-1 genes, consistent with an acute endogenous antioxidant response, a transcriptional pattern documented previously in both PTZ and kainic acid models [32,33,34,58]. This reactive upregulation, however, was insufficient to prevent the concurrent surge in oxidative stress markers (ROS, reduced SOD activity) and cytokine levels observed in our biochemical assays, reflecting the well-documented imbalance between Nrf2 activation and sustained oxidative damage in the post-ictal brain [31,32]. The significant reduction in GFAP and Iba1 transcript levels in PTZ animals treated with Morin-PLGA-CS NPs is consistent with a possible modulation of astrocytic and microglial responses. However, because these data represent mRNA abundance only, they do not confirm changes in protein expression or functional glial activation. Confirmation by immunohistochemistry or Western blotting is required [34,35]. The greater reduction of these marker mRNAs by Morin-PLGA-CS NPs than by free morin may indicate a formulation-related difference in the observed transcriptional response. However, this interpretation is speculative and requires pharmacokinetic and mechanistic studies to confirm a causal link between improved delivery and greater transcriptional anti-neuroinflammatory activity.

The substantially greater upregulation of Nrf2 and HO-1 by Morin-PLGA-CS NPs compared to free morin may further support this interpretation. HO-1 is an important regulator of various genes that code for antioxidant and anti-inflammatory proteins. Impaired HO-1 function is mechanistically linked to increased seizure susceptibility and hippocampal neuronal loss in mouse models [59,60], and HO-1 is a canonical downstream target of Nrf2 transactivation via the antioxidant response element [32,61]. The stepwise increase in transcript levels is compatible with greater pharmacodynamic effects of the nanoparticle formulation, but cannot be attributed to prolonged drug availability without pharmacokinetic data. This pattern may reflect greater Nrf2/HO-1-associated transcriptional responses than those observed with free morin. However, this remains a hypothesis that requires further investigation to establish a direct causal relationship. Collectively, the transcript-level data suggest that nanoencapsulation may contribute to stronger upregulation of the Nrf2/HO-1 antioxidant axis and may be associated with reduced glial-related transcripts. However, these observations at the mRNA level need to be validated at the protein level and in functional assays to confirm a mechanistic role [51,52,62].

The findings of our investigation suggested morin’s anticonvulsant properties, supported by several studies showing its multi-target effects on seizure pathophysiology and related neuropsychiatric issues. In this regard, the study by Abd El-Aal et al. (2022) explores the anticonvulsant and neuroprotective effects of morin using a PTZ-induced kindling model in rats [16]. Chronic PTZ administration (35 mg/kg every other day for 25 days) led to seizure acquisition, cognitive deficits, and neuronal degeneration. Morin treatment (10 mg/kg loading dose, then 5 mg/kg twice daily) notably reduced seizure scores, prolonged seizure latency, preserved CA1 neuronal viability, and improved memory. Mechanistically, morin inhibited necroptotic signaling, mitochondrial fragmentation, and the IL-6 inflammatory axis, while enhancing the Nrf2/HO-1 antioxidant pathway. Unlike their findings of increased Nrf2 and HO-1 protein levels with free morin, our nanoencapsulated morin (Morin-PLGA-CS NPs) resulted in even greater upregulation, potentially due to a different experimental setup. This observation suggests that the improved anticonvulsant efficacy may reflect a combination of formulation-related performance and morin’s previously reported antioxidant and anti-inflammatory properties. Overall, morin’s therapeutic advantage may lie in supporting endogenous antioxidant defenses, particularly enhanced by PLGA- CS nanoencapsulation. Moreover, Kandhare et al. (2018) demonstrated that morin significantly attenuated seizure onset, reduced seizure duration, and decreased mortality in rats with PTZ-induced seizures, attributing these effects to modulation of GABA and dopamine levels and restoration of Na^+^/K^+^-ATPase activity, indicating improved neuronal membrane stability [45]. Our findings supported this, as free morin prolonged seizure latency (114.5 s vs. 88.9 s in the PTZ group), and Morin-PLGA-CS NPs extended this latency to 137.2 s, a 20% improvement over free morin. Additionally, evidence showed that morin had antioxidant potential, as evidenced by reductions in malondialdehyde (MDA), nitric oxide (NO), and xanthine oxidase activity. It can enhance endogenous antioxidant defenses such as SOD and glutathione (GSH). However, the role of NO in epilepsy and seizures is complex and depends on its enzymatic source and the phase of the seizure process [63,64]. NO has a context-dependent role in epilepsy, varying by cellular source, inflammatory milieu, and seizure phase. In experimental models, NO contributes to nitro-oxidative stress, with nNOS-derived NO involved in early neuronal signaling and iNOS in sustained inflammatory injury [65,66]. Because the present study did not differentiate among NOS isoforms, the findings should be interpreted as broad attenuation of nitro-oxidative stress rather than isoform-specific modulation.

Although this study focused on inflammatory endpoints, the 50–55% reduction of IL-1β, IL-6, and TNF-α in both cortex and hippocampus is complementary to mechanistic effects. Oxidative stress and neuroinflammation are linked in seizure pathology [62]. Reactive oxygen species activate transcription factors like NF-κB, driving pro-inflammatory cytokine transcription [67,68]. Thus, the antioxidant effects reported by Kandhare et al. and the anti-inflammatory effects observed in our study likely represent aspects of a unified neuroprotective mechanism of morin, interrupting the cycle of oxidative injury and inflammatory signaling. In a PTZ-induced kindling model, El-Aal et al. (2022) demonstrated that morin reduced seizure severity and delayed kindling progression, while attenuating hippocampal neuroinflammation, oxidative stress, and neuronal injury [16]. This is relevant to our study, which assessed anxiolytic and cognitive endpoints alongside anticonvulsant activity. Their observation that morin had improved outcomes in anxiety paradigms aligns with our data, showing Morin-PLGA-CS NPs produced a 142% increase in open-arm time and a 187% increase in center time compared to vehicle control group. The consistency of these anxiolytic effects across studies might suggest that morin’s anxiolytic-like effect is robust and reproducible. The enhancement by nanoencapsulation suggests that morin’s efficacy is likely limited by pharmacokinetic factors rather than by an inherent pharmacological ceiling.

## 5. Limitations and Future Directions

Despite these encouraging outcomes, several limitations must be noted. First, the study used only adult male Wistar rats in an acute PTZ model, limiting generalization to females, sex differences, chronic epileptogenesis, or spontaneous recurrent seizures. Sex may affect seizure susceptibility, pharmacokinetics, neuroinflammatory and oxidative responses, behavioral performance, and sensitivity to sedative or anticonvulsant interventions. Therefore, these findings apply only to adult male rats under the tested conditions. Future work should include both sexes in a factorial design with adequate sample-size planning for sex, treatment, and sex-by-treatment interactions. Second, biochemical and molecular endpoints were measured at different post-PTZ time points (cytokines, SOD, ROS at 12 h; qRT-PCR and caspase-3 at 24 h) in separate cohorts, giving complementary snapshots rather than a longitudinal correlation, and qRT-PCR assessed only gene transcripts, without protein-level or histological confirmation, so pathway activation, reactive gliosis, and cellular localization remain unverified. Third, the DCFH-DA assay provided only a general oxidative-burden index without species-specific ROS identification, and the behavioral benefits in the novel object recognition test should be expanded with a broader neurocognitive battery (e.g., Morris water maze, Y-maze) plus additional motor-control measures to separate anxiety-related confounds, since reduced locomotor activity may have affected open-field and elevated-plus-maze results. Fourth, although the nanoparticles showed prolonged in vitro morin release, key pharmacokinetic and biodistribution parameters, including plasma and brain morin concentrations, blood–brain barrier transport, in vivo distribution, metabolic stability, and batch-to-batch reproducibility, were not directly measured; thus, enhanced CNS delivery and prolonged hippocampal exposure need confirmation with future studies. Fifth, long-term safety, biocompatibility, and formulation-specific stability after repeated dosing remain unaddressed. Additionally, because DLS measurements were performed immediately after reconstitution without time-dependent characterization or independent-batch analyses, conclusions about long-term formulation stability or batch-to-batch reproducibility are not supported by the current data. Overall, these limitations highlight the need for future studies to use endpoint-specific power calculations, multiplex cytokine and glial activation profiling, protein-level and histological validation, rigorous pharmacokinetic and toxicological assessments, and chronic epilepsy models before clinical consideration of Morin-PLGA-CS nanoparticles.

## 6. Conclusions

Morin-PLGA-CS NPs showed nanoscale dispersion characteristics, efficient morin loading, and prolonged in vitro release under the tested conditions. In an acute PTZ-induced seizure model, an equivalent morin dose delivered in the nanoparticle formulation produced greater effects than free morin on seizure latency, anxiety-related behavioral measures, pentobarbital-induced sleep, recognition memory performance, pro-inflammatory cytokine levels, hippocampal SOD activity, DCFH-derived fluorescence interpreted as a general oxidative-burden index, and caspase-3 activity. These outcomes were accompanied, in separate cohorts and at different post-PTZ time points, by changes in hippocampal transcript levels and biochemical markers. However, because these molecular and biochemical measures were obtained from independent cohorts at different post-PTZ time points, they should be interpreted as associated findings rather than evidence of a confirmed causal pathway. The reduction in locomotor activity also warrants caution in interpreting the behavioral data. Overall, these preclinical findings suggest that PLGA-CS nanoencapsulation may enhance the acute pharmacodynamic profile of morin in male rats. However, because the study did not include pharmacokinetic or brain-distribution measurements, these findings cannot be interpreted as evidence of enhanced CNS delivery. Confirmation of brain exposure, protein-level mechanisms, formulation-specific safety, and efficacy in chronic epilepsy models is still required before translational conclusions can be drawn.

## Figures and Tables

**Table 1:** Primer sequences used for qRT-PCR analysis.

Primer	Sequence
*HO-1*	F 5′-CACCGGATAGAGCGCGACAG-3′ R 5′-GGTAGGTGAAGGACTTGGCTGG-3′
*Nrf2*	F 5′-TTGTAGATGACCATGAGTCGC-3′ R 5′-ACTTCCAGGGGCACTGTCTA-3′
*GFAP*	F 5′-TCGAGATCGCCACCTACAG-3′ R 5′-GTCTGTACAGGAATGGTGATGC-3′
*Iba1*	F 5′-GGATTTGCAGGGAGGAAAG-3′ R 5′-TGGGATCATCGAGGAATTG-3′
*GAPDH*	F 5′-AGTGCCAGCCTCGTCTCATA-3′ R 5′-GATGGTGATGGGTTTCCCGT-3′

**Table 2:** FTIR peak assignments of morin, PLGA-CS NPs, and morin-loaded PLGA-CS NPs.

Functional Group/Assignment	Morin (cm^−1^)	PLGA-CS NPs (cm^−1^)	Morin-Loaded PLGA-CS NPs (cm^−1^)	Observed Shift/Change
νO–H/νN–H (hydrogen-bonded)	3405 (broad)	3440 (broad)	3420 (broad, broader)	Shift: +15 cm^−1^, broadening
νC–H (aliphatic)	2900	2993, 2930, 2875	2950, 2928	Slight broadening
νC=O (ester) (PLGA diagnostic)	—	1750	1749	Slight intensity reduction
νC=O (conjugated) (morin)	1661	—	1645 (overlapped with amide I)	Shift: −16 cm^−1^
Amide I (νC=O) (CS)	—	1648	1645 (overlapped)	Overlap with morin C=O
νC=C (aromatic) (morin)	1612, 1580, 1515	—	1600, 1580, 1515 (reduced intensity)	Intensity reduction
Amide II (δN–H + νC–N) (CS)	—	1588, 1560	1560, 1550	Slight shift
δC–H/δO–H	1459, 1445, 1376	1452, 1416, 1384	1450, 1376 (reduced)	Intensity reduction
νC2′–OH (morin-specific)	1308	—	1308 (very weak)	Significant intensity reduction
νC–O–C (ether, morin)	1253	—	1253 (weak/shoulder)	Marked intensity reduction
νC–O–C (glycosidic) (CS)	—	1150, 1148	1150–1148	Retained
νC–O (saccharide/CS)	—	1087, 1061, 1026	1080–1026 (broad)	Broadening

## Data Availability

The datasets generated and analyzed during the current study are available from the corresponding author upon reasonable request.

## Supplementary Materials

The following supporting information can be downloaded at https://www.mdpi.com/article/10.3390/pharmaceutics18091170/s1. Supplementary Figure S1. Standard curve obtained from serial p-nitroaniline (pNA) standards ranging from 0 to 200 µM. Absorbance at 405 nm showed a linear relationship with pNA concentration and was used to calculate caspase-3 enzyme activity through linear regression analysis. For comparative analysis, caspase-3 activity values were normalized to the non-PTZ vehicle-treated control and expressed as relative fold change, with the non-PTZ vehicle-treated control assigned a value of 1.0. Supplementary Figure S2. Scanning electron microscopy (SEM) image of polymeric nanocarriers composed of poly (lactic-co-glycolic acid) (PLGA) and chitosan (CS) (PLGA-CS NPs), revealing their characteristic morphology and nanoscale dimensions. The nanoparticles show the representative morphology of the PLGA-CS nanoparticles.

## Abbreviations

The following abbreviations are used in this manuscript:

ANOVA	Analysis of variance
APC	Article processing charge
ASM	Antiseizure medication
BBB	Blood–brain barrier
BCA	Bicinchoninic acid
BDC	Biodiagnostic Center
BDNF	Brain-derived neurotrophic factor
BHT	Butylated hydroxytoluene
CNS	Central nervous system
CS	Chitosan
DCF	Dichlorofluorescein
DCFH-DA	2′,7′-Dichlorodihydrofluorescein diacetate
DCM	Dichloromethane
DEVD-pNA	Asp-Glu-Val-Asp-p-nitroanilide
DI	Discrimination index
DLS	Dynamic light scattering
DMSO	Dimethyl sulfoxide
DL%	Drug loading
DTT	Dithiothreitol
EDTA	Ethylenediaminetetraacetic acid
EE%	Encapsulation efficiency
ELISA	Enzyme-linked immunosorbent assay
EPM	Elevated-plus maze
FDA	U.S. Food and Drug Administration
FTIR	Fourier-transform infrared spectroscopy
GABA	Gamma-aminobutyric acid
GABA_A_	Gamma-aminobutyric acid type A
GAPDH	Glyceraldehyde-3-phosphate dehydrogenase
GFAP	Glial fibrillary acidic protein
GSH	Glutathione
HO-1	Heme oxygenase-1
IACUC	Institutional Animal Care and Use Committee
Iba1	Ionized calcium-binding adapter molecule 1
iNOS	Inducible nitric oxide synthase
IL-1β	Interleukin-1 beta
IL-6	Interleukin-6
KBr	Potassium bromide
LC-MS/MS	Liquid chromatography–tandem mass spectrometry
LPS	Lipopolysaccharide
MAPKs	Mitogen-activated protein kinases
MDA	Malondialdehyde
mTORC1	Mechanistic target of rapamycin complex 1
NF-κB	Nuclear factor kappa B
nNOS	Neuronal nitric oxide synthase
NO	Nitric oxide
NOR	Novel object recognition
NPs	Nanoparticles
Nrf2	Nuclear factor erythroid 2-related factor 2
OFT	Open-field test
PBS	Phosphate-buffered saline
PDI	Polydispersity index
PI3K/Akt	Phosphoinositide 3-kinase/protein kinase B
PKA	Protein kinase A
PLGA	Poly(lactic-co-glycolic acid)
PTZ	Pentylenetetrazol
PVA	Polyvinyl alcohol
qRT-PCR	Quantitative real-time polymerase chain reaction
RFU	Relative fluorescence units
RIPA	Radioimmunoprecipitation assay
ROS	Reactive oxygen species
SD	Standard deviation
SEM	Scanning electron microscopy
SOD	Superoxide dismutase
SPSS	Statistical Package for the Social Sciences
TNF-α	Tumor necrosis factor-alpha
UV–Vis	Ultraviolet–visible
WST-1	Water-soluble tetrazolium salt-1
cDNA	Complementary DNA
DNA	Deoxyribonucleic acid
DNase	Deoxyribonuclease
RNA	Ribonucleic acid
MW	Molecular weight
MWCO	Molecular weight cut-off
R^2^	Coefficient of determination
Ct	Cycle threshold
OD	Optical density
rpm	Revolutions per minute
GenAI	Generative artificial intelligence
CC BY	Creative Commons Attribution
TNFR-1	Tumor necrosis factor receptor 1
RIPK1	Receptor-interacting serine/threonine-protein kinase 1
MLKL	Mixed lineage kinase domain-like protein
PGAM5	Phosphoglycerate mutase family member 5
Drp-1	Dynamin-related protein 1
JAK2	Janus kinase 2
STAT3	Signal transducer and activator of transcription 3
ARE	Antioxidant response element
Keap-1	Kelch-like ECH-associated protein 1
Nox-2	NADPH oxidase 2
Na^+^/K^+^-ATPase	Sodium–potassium adenosine triphosphatase

## References

[1] Eslamian M., Shafiei H., Mojahed F., Bahreini A. (2024). Prevalence of epilepsy in children and adolescents worldwide: A literature overview. Health Prov..

[2] Beghi E. (2020). The Epidemiology of Epilepsy. Neuroepidemiology.

[3] Giourou E., Stavropoulou-Deli A., Giannakopoulou A., Kostopoulos G.K., Koutroumanidis M. (2015). Introduction to epilepsy and related brain disorders. Cyberphysical Systems for Epilepsy and Related Brain Disorders: Multi-Parametric Monitoring and Analysis for Diagnosis and Optimal Disease Management.

[4] Beghi E. (2016). Addressing the burden of epilepsy: Many unmet needs. Pharmacol. Res..

[5] Beleza P. (2009). Refractory epilepsy: A clinically oriented review. Eur. Neurol..

[6] Mao J., Takahashi K., Cheng M., Xu C., Boca A., Song Y., Dandurand A. (2024). Real-world anti-seizure treatment and adverse events among individuals living with drug-resistant focal epilepsy in the United States. Epilepsia Open.

[7] Sharma A.K. (2025). Beyond the Label: A Literature Review on the Neurotoxic Effects of Everyday Medications. Master’s Thesis.

[8] Riva A., Golda A., Balagura G., Amadori E., Vari M.S., Piccolo G., Iacomino M., Lattanzi S., Salpietro V., Minetti C. (2021). New Trends and Most Promising Therapeutic Strategies for Epilepsy Treatment. Front. Neurol..

[9] Araujo-Filho H.G., Quintans-Junior L.J., Barreto A.S., Almeida J.R., Barreto R.S., Quintans J.S. (2016). Neuroprotective Effect of Natural Products on Peripheral Nerve Degeneration: A Systematic Review. Neurochem. Res..

[10] Saboon, Chaudhari S.K., Arshad S., Amjad M.S., Akhtar M.S. (2019). Natural compounds extracted from medicinal plants and their applications. Natural Bio-Active Compounds: Volume 1: Production and Applications.

[11] Panche A.N., Diwan A.D., Chandra S.R. (2016). Flavonoids: An overview. J. Nutr. Sci..

[12] Kumar S., Raghuvanshi A., Kumar A., Monga V. (2026). Morin: A Comprehensive Review of Its Sources, Nutraceutical Prospects, Chemistry, and Pharmacological Insights. Phytother. Res..

[13] Jain S., Yadav Y., Sharma M. (2025). Morin: A powerful potential flavonoid for human. J. Drug Deliv. Ther..

[14] Lem F.F., Jiunn Herng Lee D., Chee F.T. (2023). Pharmacological insights into morin: Therapeutic applications and future perspectives. Handbook of Dietary Flavonoids.

[15] Noor S.M., Reddy D.H., Srikanth Y., Viswanadh M.K., Dumala N., Chakravarthy G., Nalluri B.N., Naryanarao A., Duguluri S., Yadagiri G. (2026). Morin hydrate: A comprehensive review on therapeutic potential in treating neurological diseases. Nutr. Neurosci..

[16] El-Aal A., Sarah A., El-Abhar H.S., Abulfadl Y.S. (2022). Morin offsets PTZ-induced neuronal degeneration and cognitive decrements in rats: The modulation of TNF-α/TNFR-1/RIPK1, 3/MLKL/PGAM5/Drp-1, IL-6/JAK2/STAT3/GFAP and Keap-1/Nrf-2/HO-1 trajectories. Eur. J. Pharmacol..

[17] Rabidas S.S., Prakash C., Tyagi J., Suryavanshi J., Kumar P., Bhattacharya J., Sharma D. (2023). A Comprehensive Review on Anti-Inflammatory Response of Flavonoids in Experimentally-Induced Epileptic Seizures. Brain Sci..

[18] Ravizza T., Scheper M., Di Sapia R., Gorter J., Aronica E., Vezzani A. (2024). mTOR and neuroinflammation in epilepsy: Implications for disease progression and treatment. Nat. Rev. Neurosci..

[19] Lee J.M., Hong J., Moon G.J., Jung U.J., Won S.Y., Kim S.R. (2018). Morin Prevents Granule Cell Dispersion and Neurotoxicity via Suppression of mTORC1 in a Kainic Acid-induced Seizure Model. Exp. Neurobiol..

[20] Patel N., Patel P., Thummar D. (2024). Design and Development of Self-nanoemulsifying Drug Delivery System (SNEDDS) with Morin Hydrate to Enhance Nimodipine’s oral Bioavailability and Pharmacokinetics. Nano Biomed. Eng..

[21] Sharma D., Singh M., Kumar P., Vikram V., Mishra N. (2017). Development and characterization of morin hydrate loaded microemulsion for the management of Alzheimer’s disease. Artif. Cells Nanomed. Biotechnol..

[22] Alabrahim O.A.A., Azzazy H.M.E. (2022). Polymeric nanoparticles for dopamine and levodopa replacement in Parkinson’s disease. Nanoscale Adv..

[23] Cacciatore I., Ciulla M., Fornasari E., Marinelli L., Di Stefano A. (2016). Solid lipid nanoparticles as a drug delivery system for the treatment of neurodegenerative diseases. Expert. Opin. Drug Deliv..

[24] Tosi G., Bortot B., Ruozi B., Dolcetta D., Vandelli M.A., Forni F., Severini G.M. (2013). Potential use of polymeric nanoparticles for drug delivery across the blood-brain barrier. Curr. Med. Chem..

[25] Pinto M., Silva V., Barreiro S., Silva R., Remiao F., Borges F., Fernandes C. (2022). Brain drug delivery and neurodegenerative diseases: Polymeric PLGA-based nanoparticles as a forefront platform. Ageing Res. Rev..

[26] Abbas H., Sayed N.S.E., Youssef N.A.H.A., Gaafar P.M.E., Mousa M.R., Fayez A.M., Elsheikh M.A. (2022). Novel Luteolin-Loaded Chitosan Decorated Nanoparticles for Brain-Targeting Delivery in a Sporadic Alzheimer’s Disease Mouse Model: Focus on Antioxidant, Anti-Inflammatory, and Amyloidogenic Pathways. Pharmaceutics.

[27] Bhattacharjee A., Savargaonkar A.V., Tahir M., Sionkowska A., Popat K.C. (2024). Surface modification strategies for improved hemocompatibility of polymeric materials: A comprehensive review. RSC Adv..

[28] Vijayakumar M.R., Kosuru R., Singh S.K., Prasad C.B., Narayan G., Muthu M.S., Singh S. (2016). Resveratrol loaded PLGA: D-α-tocopheryl polyethylene glycol 1000 succinate blend nanoparticles for brain cancer therapy. RSC Adv..

[29] Hosseini S.M., Mazinani S., Abdouss M., Kalhor H., Kalantari K., Amiri I.S., Ramezani Z. (2022). Designing chitosan nanoparticles embedded into graphene oxide as a drug delivery system. Polym. Bull..

[30] Smith J., Wood E., Dornish M. (2004). Effect of chitosan on epithelial cell tight junctions. Pharm. Res..

[31] Carmona-Aparicio L., Perez-Cruz C., Zavala-Tecuapetla C., Granados-Rojas L., Rivera-Espinosa L., Montesinos-Correa H., Hernandez-Damian J., Pedraza-Chaverri J., Sampieri A., Coballase-Urrutia E. (2015). Overview of Nrf2 as Therapeutic Target in Epilepsy. Int. J. Mol. Sci..

[32] Sandouka S., Shekh-Ahmad T. (2021). Induction of the Nrf2 Pathway by Sulforaphane Is Neuroprotective in a Rat Temporal Lobe Epilepsy Model. Antioxidants.

[33] Sandouka S., Saadi A., Singh P.K., Olowe R., Shekh-Ahmad T. (2023). Nrf2 is predominantly expressed in hippocampal neurons in a rat model of temporal lobe epilepsy. Cell Biosci..

[34] Shapiro L.A., Wang L., Ribak C.E. (2008). Rapid astrocyte and microglial activation following pilocarpine-induced seizures in rats. Epilepsia.

[35] Zakharova M.V., Kovalenko A.A., Zubareva O.E., Schwarz A.P., Postnikova T.Y., Trofimova A.M., Zaitsev A.V. (2024). Pentylenetetrazole-Induced Seizures Cause Short-Term Changes in the Phenotype of Microglial and Astroglial Cells in the Hippocampus and Temporal Cortex of Young Male Wistar Rats. J. Neurosci. Res..

[36] Jung J.S., Choi M.J., Lee Y.Y., Moon B.I., Park J.S., Kim H.S. (2017). Suppression of Lipopolysaccharide-Induced Neuroinflammation by Morin via MAPK, PI3K/Akt, and PKA/HO-1 Signaling Pathway Modulation. J. Agric. Food Chem..

[37] Yang F., Cabe M., Nowak H.A., Langert K.A. (2022). Chitosan/poly(lactic-co-glycolic)acid Nanoparticle Formulations with Finely-Tuned Size Distributions for Enhanced Mucoadhesion. Pharmaceutics.

[38] Gould T.D., Dao D.T., Kovacsics C.E. (2009). The open field test. Mood and Anxiety Related Phenotypes in Mice: Characterization Using Behavioral Tests.

[39] Rodgers R.J., Dalvi A. (1997). Anxiety, defence and the elevated plus-maze. Neurosci. Biobehav. Rev..

[40] Abdolmaleki A., Moghimi A., Ghayour M.B., Rassouli M.B. (2016). Evaluation of neuroprotective, anticonvulsant, sedative and anxiolytic activity of citicoline in rats. Eur. J. Pharmacol..

[41] Luttjohann A., Fabene P.F., van Luijtelaar G. (2009). A revised Racine’s scale for PTZ-induced seizures in rats. Physiol. Behav..

[42] Lueptow L.M. (2017). Novel Object Recognition Test for the Investigation of Learning and Memory in Mice. J. Vis. Exp..

[43] Klein P., Krauss G.L., Steinhoff B.J., Devinsky O., Sperling M.R. (2023). Failure to use new breakthrough treatments for epilepsy. Epilepsia.

[44] Olsen R.W. (2020). Convulsant and anticonvulsant drug receptor binding. Receptor Binding in Drug Research.

[45] Kandhare A.D., Mukherjee A.A., Bodhankar S.L. (2018). Anti-epileptic effect of morin against experimental pentylenetetrazol-induced seizures via modulating brain monoamines and oxidative stress. Asian Pac. J. Trop. Biomed..

[46] Ben-Azu B., Aderibigbe A.O., Ajayi A.M., Eneni A.O., Umukoro S., Iwalewa E.O. (2018). Involvement of GABAergic, BDNF and Nox-2 mechanisms in the prevention and reversal of ketamine-induced schizophrenia-like behavior by morin in mice. Brain Res. Bull..

[47] Sales L.S., Hewitt B., Muchova M., Brighenti F.L., Kuehne S.A., Grant M.M., Milward M.R. (2025). Anti-inflammatory, antioxidant, and antimicrobial evaluation of morin. Arch. Oral Biol..

[48] Costa E., Guidotti A., Toffano G. (1978). Molecular mechanisms mediating the action of diazepam on GABA receptors. Br. J. Psychiatry.

[49] Alonso M., Barcia E., Gonzalez J.F., Montejo C., Garcia-Garcia L., Villa-Hermosilla M.C., Negro S., Fraguas-Sanchez A.I., Fernandez-Carballido A. (2022). Functionalization of Morin-Loaded PLGA Nanoparticles with Phenylalanine Dipeptide Targeting the Brain. Pharmaceutics.

[50] Wu L., Qin Y., Yuan H., Zhu Y., Hu A. (2022). Anti-inflammatory and neuroprotective effects of insulin-like growth factor-1 overexpression in pentylenetetrazole (PTZ)-induced mouse model of chronic epilepsy. Brain Res..

[51] Obydah W., Abouelnaga A.F., Abass M., Saad S., Yehia A., Ammar O.A., Badawy A.M., Ibrahim M.M., Hussein A.M. (2023). Possible Role of Oxidative Stress and Nrf2/HO-1 Pathway in Pentylenetetrazole-induced Epilepsy in Aged Rats. Rep. Biochem. Mol. Biol..

[52] Manavi M.A., Mohammad Jafari R., Shafaroodi H., Dehpour A.R. (2025). The Keap1/Nrf2/ARE/HO-1 axis in epilepsy: Crosstalk between oxidative stress and neuroinflammation. Int. Immunopharmacol..

[53] Kapur J. (2000). Hippocampal neurons express GABAA receptor insensitive to diazepam in hyperexcitable conditions. Epilepsia.

[54] Olsen R.W., Wallner M., Rogawski M.A., Noebels J.L., Avoli M., Rogawski M.A., Vezzani A., Delgado-Escueta A.V. (2024). GABAA receptors, seizures, and epilepsy. Jasper’s Basic Mechanisms of the Epilepsies.

[55] Elsayed H.R.H., Rabei M.R., Elshaer M.M.A., El Nashar E.M., Alghamdi M.A., Al-Qahtani Z., Nabawy A. (2023). Suppression of neuronal apoptosis and glial activation with modulation of Nrf2/HO-1 and NF-kB signaling by curcumin in streptozotocin-induced diabetic spinal cord central neuropathy. Front. Neuroanat..

[56] Winiarska-Mieczan A., Baranowska-Wojcik E., Kwiecien M., Grela E.R., Szwajgier D., Kwiatkowska K., Kiczorowska B. (2020). The Role of Dietary Antioxidants in the Pathogenesis of Neurodegenerative Diseases and Their Impact on Cerebral Oxidoreductive Balance. Nutrients.

[57] Rice M.E. (2000). Ascorbate regulation and its neuroprotective role in the brain. Trends Neurosci..

[58] Wang W., Wang W.P., Zhang G.L., Wu Y.F., Xie T., Kan M.C., Fang H.B., Wang H.C. (2013). Activation of Nrf2-ARE signal pathway in hippocampus of amygdala kindling rats. Neurosci. Lett..

[59] Liang L.P., Patel M.N. (2004). Mitochondrial oxidative stress and increased seizure susceptibility in Sod2(−/+) mice. Free Radic. Biol. Med..

[60] Fulton R.E., Pearson-Smith J.N., Huynh C.Q., Fabisiak T., Liang L.P., Aivazidis S., High B.A., Buscaglia G., Corrigan T., Valdez R. (2021). Neuron-specific mitochondrial oxidative stress results in epilepsy, glucose dysregulation and a striking astrocyte response. Neurobiol. Dis..

[61] Goodfellow M.J., Borcar A., Proctor J.L., Greco T., Rosenthal R.E., Fiskum G. (2020). Transcriptional activation of antioxidant gene expression by Nrf2 protects against mitochondrial dysfunction and neuronal death associated with acute and chronic neurodegeneration. Exp. Neurol..

[62] Parsons A.L.M., Bucknor E.M.V., Castroflorio E., Soares T.R., Oliver P.L., Rial D. (2022). The Interconnected Mechanisms of Oxidative Stress and Neuroinflammation in Epilepsy. Antioxidants.

[63] Alkhamees O.A. (2013). Morin a flavonoid exerts antioxidant potential in streptozotocin-induced hepatotoxicity. Br. J. Pharmacol. Toxicol..

[64] Subash S., Subramanian P. (2009). Morin a flavonoid exerts antioxidant potential in chronic hyperammonemic rats: A biochemical and histopathological study. Mol. Cell Biochem..

[65] Sharma S., Puttachary S., Thippeswamy T. (2019). Glial source of nitric oxide in epileptogenesis: A target for disease modification in epilepsy. J. Neurosci. Res..

[66] Banach M., Piskorska B., Czuczwar S.J., Borowicz K.K. (2011). Nitric oxide, epileptic seizures, and action of antiepileptic drugs. CNS Neurol. Disord. Drug Targets.

[67] Alenazi F., Moursi S., Mahmoud M.R., Shahid S.M.A., Khatoon F., Shahid Khan M., Khan M.A., Alam M.J., Saleem M., Syed Khaja A.S. (2024). Withaferin A alleviates inflammation in animal models of arthritis by inhibiting the NF-kappaB pathway and cytokine release. Chem. Biol. Interact..

[68] Xie C., Ma H., Shi Y., Li J., Wu H., Wang B., Shao Z., Huang C., Chen J., Sun L. (2021). Cardamonin protects nucleus pulposus cells against IL-1beta-induced inflammation and catabolism via Nrf2/NF-kappaB axis. Food Funct..

